# Retinoic Acid Promotes Neuronal Differentiation While Increasing Proteins and Organelles Related to Autophagy

**DOI:** 10.3390/ijms26041691

**Published:** 2025-02-16

**Authors:** Gloria Lazzeri, Paola Lenzi, Giulia Signorini, Sara Raffaelli, Elisa Giammattei, Gianfranco Natale, Riccardo Ruffoli, Francesco Fornai, Michela Ferrucci

**Affiliations:** 1 Department of Translational Research and New Technologies in Medicine and Surgery, University of Pisa, 56126 Pisa, Italy; gloria.lazzeri@unipi.it (G.L.); paola.lenzi@unipi.it (P.L.); signorinigiulia01@gmail.com (G.S.); saraffaelli29@gmail.com (S.R.); elisagiammattei288@gmail.com (E.G.); gianfranco.natale@unipi.it (G.N.); riccardo.ruffoli@unipi.it (R.R.); francesco.fornai@unipi.it (F.F.); 2 IRCCS-Istituto di Ricovero e Cura a Carattere Scientifico, Neuromed, 86077 Pozzilli, Italy

**Keywords:** ultrastructural morphology, cell morphometry, immunoelectron microscopy, nestin, NeuN, Beclin 1, LC3, autophagy vacuoles, 3-methiladenine, rapamycin

## Abstract

Retinoic acid (RA) is commonly used to differentiate SH-SY5Y neuroblastoma cells. This effect is sustained by a specific modulation of gene transcription, leading to marked changes in cellular proteins. In this scenario, autophagy may be pivotal in balancing protein synthesis and degradation. The present study analyzes whether some autophagy-related proteins and organelles are modified during RA-induced differentiation of SH-SY5Y cells. RA-induced effects were compared to those induced by starvation. SH-SY5Y cells were treated with a single dose of 10 µM RA or grown in starvation, for 3 days or 7 days. After treatments, cells were analyzed at light microscopy and transmission electron microscopy to assess cell morphology and immunostaining for specific markers (nestin, βIII-tubulin, NeuN) and some autophagy-related proteins (Beclin 1, LC3). We found that both RA and starvation differentiate SH-SY5Y cells. Specifically, cell differentiation was concomitant with an increase in autophagy proteins and autophagy-related organelles. However, the effects of a single dose of 10 μM RA persist for at least 7 days, while prolonged starvation produces cell degeneration and cell loss. Remarkably, the effects of RA are modulated in the presence of autophagy inhibitors or stimulators. The present data indicate that RA-induced differentiation is concomitant with an increased autophagy.

## 1. Introduction

The SH-SY5Y neuroblastoma cell line is an immortalized subclone of the parental neuroblastoma cell line SK-N-SH, which was established in 1970 from a bone marrow biopsy of a metastatic tissue [1]. As immature neoplastic cells derived from the neural crests, SH-SY5Y cells retain some properties of undifferentiated stem-like cells, which is consistent with a high proliferation rate [2]. On the other hand, these cells express some proteins specific for catecholamine-producing cells, such as tyrosine hydroxylase and dopamine-β-hydroxylase, which witnesses their catecholamine lineage [3,4,5]. Therefore, SH-SY5Y cells are commonly used as in vitro model for catecholamine neurodegeneration and neurotoxicity [2,6,7,8,9,10].

When cultured under specific medium conditions, SH-SY5Y cells undergo a phenotypic shift from undifferentiated neuroblastoma cells into mature neuron-like cells. Such a shift may be induced by adding retinoic acid (RA) to the cell medium [11,12].

In fact, RA interacts with two classes of nuclear hormone receptors: the RA receptors and the retinoic X receptors [13], which belong to the steroid–thyroid–vitamin D superfamily [14,15]. Upon activation by RA, these receptors bind to specific DNA sequences containing RA response elements and modulate gene expression.

This generates synthesis of new proteins [16,17], some of which promote cell differentiation by epigenetic mechanisms [18,19,20,21]. For instance, RA increases the effects of brain-derived neurotrophic factor and nerve growth factor, which seem to participate in RA-induced cell differentiation [11,19,22,23].

Such a marked variation in gene expression is believed to involve some proteins which are related to the autophagy pathway [24,25]. In fact, autophagy is an evolutionarily conserved lysosomal-dependent degradative pathway which is critical for protein and organelle turn-over [26,27,28,29,30,31].

In particular, by removing misfolded proteins and damaged organelles, such as mitochondria (mitophagy) [32], endoplasmic reticulum (ER, ER-phagy) [33], and Golgi apparatus (Golgi-phagy) [26,34,35,36,37], autophagy enables the recycling of primary cell components and preserves cell homeostasis under both physiological and stressful conditions [38,39,40,41].

Autophagy also plays a key role in cell development and differentiation [42,43,44,45,46,47,48,49]. It is involved in various developmental processes, ranging from the oocyte-to-embryo transition to post-embryonic development, including neuronal development [47,48]. Specifically, autophagy is essential for the establishment of proper neuronal morphology, connectivity, and survival [50,51,52,53,54,55]. Moreover, as post-mitotic and long-lived cells, neurons depend on autophagy for their entire lifespan [54]. In line with this, defective autophagy is associated with a variety of neurodevelopmental and neurodegenerative disorders [56,57,58,59,60,61,62,63,64].

During autophagy, proteins and organelles are segregated into specialized organelles, named autophagosomes, which are the hallmark autophagy organelles [65,66,67]. Autophagy begins with the formation of the phagophore, an open membranous structure, which sequesters the cargo targeted for autophagy degradation. Then, the phagophore transforms into a closed double-membraned vacuole, the autophagosome, which finally fuses with the lysosome to form the autophagolysosome, where the cargo degradation occurs [67,68].

To validate an intriguing parallelism between an increase in autophagy-related structures and cell differentiation, in the present study, we assessed concomitantly RA-induced cell differentiation and a possible increase in some key autophagy proteins and autophagy-related organelles. The potential concomitancy between cell differentiation and autophagy perturbation was further validated by measuring cell proteins and organelles following starvation, which is known to induce autophagy and produce cell differentiation [31,69,70]. Finally, we investigated the effects of the autophagy inhibitor 3-methiladenine (3-MA) and the autophagy stimulator rapamycin on RA-induced cell differentiation by using light microscopy and transmission electron microscopy (TEM), which also allows to measure stoichiometry of autophagy-related proteins in situ within autophagy-related organelles. Our results show that RA induces neuronal differentiation of SH-SY5Y cells by increasing specific autophagy proteins and autophagy-related structures.

## 2. Results

### 2.1. Retinoic Acid Reduces SH-SY5Y Cell Amounts

**Figure 1:**
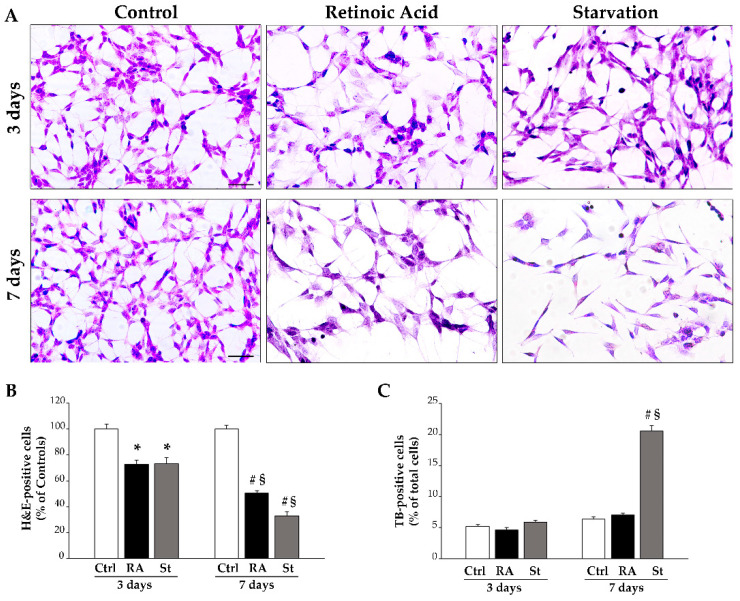
RA and starvation time-dependently reduce SH-SY5Y cell number. (**A**) Representative pictures of SH-SY5Y cells stained with hematoxylin and eosin (H&E) in control conditions and at 3 days or 7 days following treatment with RA or starvation. The graphs report (**B**) the count of H&E-stained cells and (**C**) TB-positive cells in each experimental condition. Ctrl, controls; RA, retinoic acid; St, starvation. Values are given as mean percentage ± S.E.M. from three independent experiments. * *p* < 0.05 compared with controls; ^#^ *p* < 0.05 compared with other groups; ^§^ *p* < 0.05 compared with 3 days. Scale bar = 62 μm.

Representative pictures of SH-SY5Y cells show that treatment with RA or starvation for either 3 days or 7 days produces a reduction in cells stained with hematoxylin and eosin (H&E) compared with controls (Figure 1A). This effect is confirmed by the count of H&E-stained cells reported in the graph (Figure 1B).

To evaluate the contribution of cell death in producing such a reduction in cell number, SH-SY5Y cells were stained with trypan blue (TB), which stains damaged cells. The percentage of TB-positive cells following RA or starvation was reported in the graph (Figure 1C), which shows that RA does not change the percentage of TB-positive cells compared with controls (Figure 1C). In contrast, starvation, when protracted for 7 days, causes a significant increase in the percentage of TB-positive cells compared with controls (Figure 1C).

**Figure 2:**
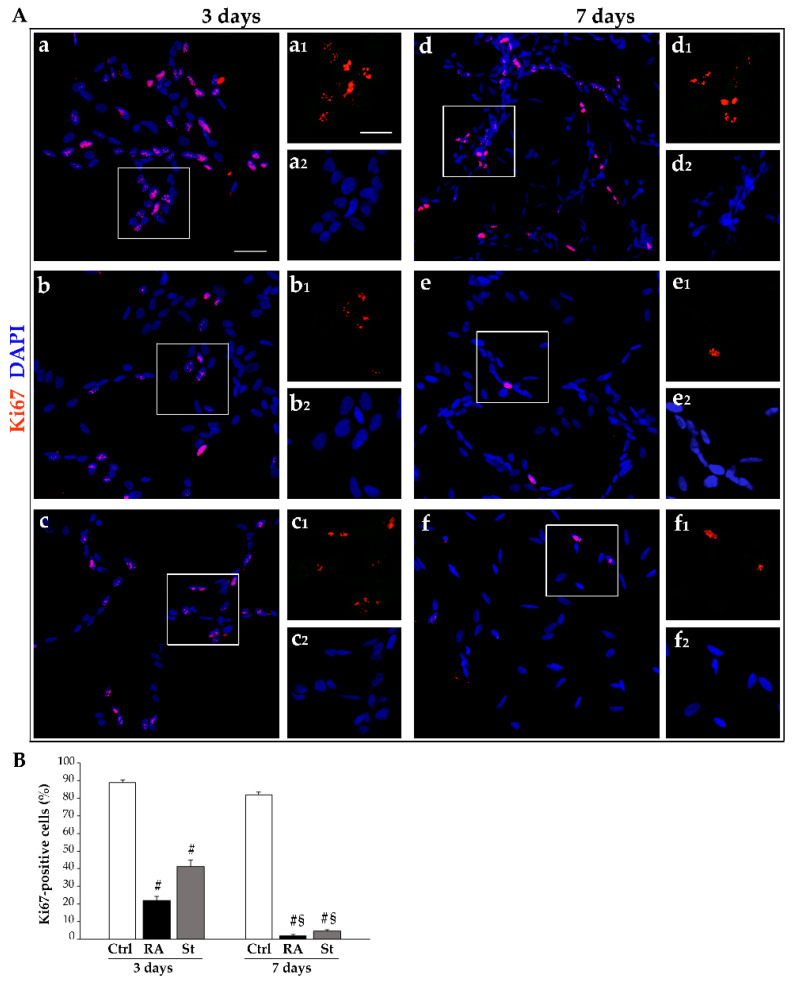
RA and starvation time-dependently inhibit SH-SY5Y cell proliferation. (**A**) Representative immunofluorescence for the Ki67 protein merged with the fluorescent nuclear dye 4′,6-diamidin-2-fenilindolo (DAPI) in (**a**,**d**) controls and (**b**,**e**) following treatment with RA or (**c**,**f**) starvation at (**a**–**c**) 3 days and (**d**–**f**) 7 days. For each experimental condition, inserts correspond to the high magnification of the squared area showing Ki67 (red, **a1**–**f1**) and DAPI (blue, **a2**–**f2**). (**B**) The graph reports the count of Ki67-immunofluorescent cells. Ctrl, controls; RA, retinoic acid; St, starvation. Values are given as mean percentage ± S.E.M. from three independent experiments. ^#^ *p* < 0.05 compared with other groups; ^§^ *p* < 0.05 compared with 3 days. Scale bars = (**a**–**f**) 7 μm; (inserts) 4 μm.

Interestingly, evaluation of cell proliferation through anti-Ki67 immunofluorescence shows that RA-treated cells develop a progressive decrease in Ki67 immunostaining. Such an effect occurs similarly following 3 days and 7 days of starvation. This is shown in the representative pictures in Figure 2A, while the count of the percentage of Ki67-positive cells is reported in the graph in Figure 2B.

These data, together with the fact that, after treatment with RA, the amount of TB-positive cells does not change (Figure 1C), suggest that, in RA-treated cells, the decrease in cell number may be entirely due to a decrease in the proliferation rate; meanwhile, following 7 days of starvation, the decrease in cell number is significantly related to altered cell integrity, which adds on a suppression of cell proliferation.

### 2.2. Retinoic Acid Changes Cell Shape and Size

The effects of RA and starvation on cell morphology were analyzed in H&E-stained SH-SY5Y cells.

**Figure 3:**
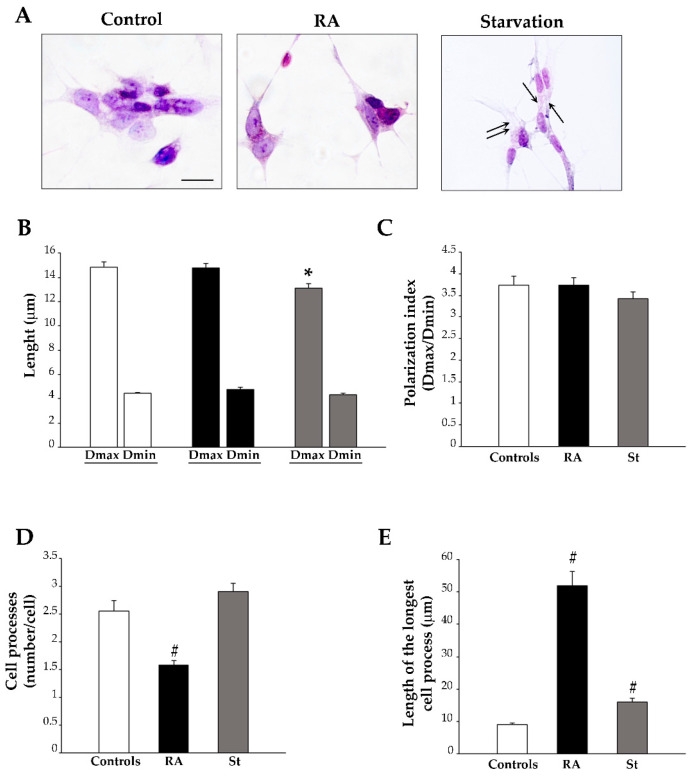
Morphological changes induced by RA and starvation in SH-SY5Y cells at 3 days. (**A**) Representative images of hematoxylin and eosin (H&E)-stained cells from controls, RA-treated cells, or starved cells at 3 days. Arrows indicate some vacuoles within starved cells. The graphs report the following findings: (**B**) the maximum and minimum cell body diameters (Dmax and Dmin, respectively); (**C**) the polarization index (Dmax/Dmin); (**D**) the number of cell processes; (**E**) the length of the longest cell process. RA, retinoic acid; St, starvation. Values are given as mean ± S.E.M. from *n* = 100 cells per experimental group obtained in three independent experiments. * *p* < 0.05 compared with controls; ^#^ *p* < 0.05 compared with other groups. Scale bar = 18 μm.

At 3 days, vehicle-treated (control) cells typically exhibit a stellate shape, due to multiple short processes. A large hematoxylin-positive nucleus is surrounded by a pale cytosol (Figure 3A). Following 3 days of RA exposure, cell shape becomes ovoid-like, and both nucleus and cytosol become intensely stained with hematoxylin. A few filiform cell processes develop, which are longer in shape compared with those observed in control cells (Figure 3A). Following 3 days of starvation, cells develop a shape which is reminiscent of that induced by RA, where the nucleus is intensely stained with hematoxylin and some filiform cell processes rise from the cell. Differing from RA, the cytosol is less homogeneous, since small pale vacuoles are present (Figure 3A). The graphs in Figure 3B–E report quantitative measurements of cell morphology, consisting in maximum and minimum cell diameters (Dmax and Dmin, respectively) (Figure 3B), along with their ratio (polarization index, Figure 3C) and the number and length of the cell processes (Figure 3D,E, respectively). These data demonstrate that RA and starvation differently affect SH-SY5Y cell morphology. In fact, at 3 days, starved cells possess a reduced Dmax compared with controls (Figure 3B); meanwhile, in RA-treated cells, the cell body size is not changed compared with that of controls (Figure 3B). The measure of the polarization index reveals that, despite the changes in the cell body shape and/or size, neither RA nor starvation modify this parameter after 3 days of treatment compared with controls (Figure 3C). Moreover, in RA-treated cells, the number of processes per cell is significantly reduced compared with both control and starved cells (Figure 3D). As a further effect, although the length of the longest cell process increases in both starved and RA-treated cells compared with controls, such an increase is significantly higher in RA-treated cells compared with starved cells (Figure 3E). These findings show that, at 3 days, RA reduces the number while markedly increasing the length of cell processes, whereas starvation alters the structure of the cell (at least concerning H&E staining) and moderately increases the length of the processes.

**Figure 4:**
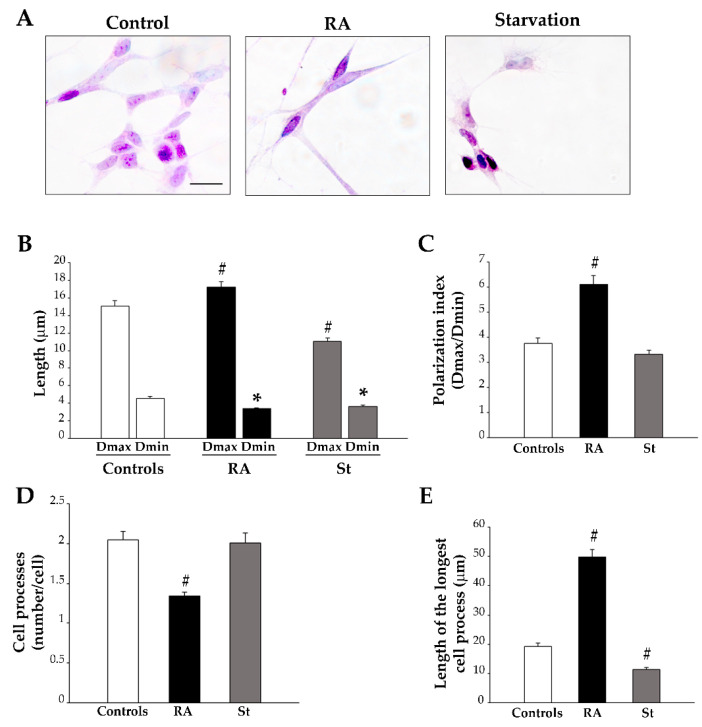
Morphological changes induced by RA and starvation in SH-SY5Y cells at 7 days. (**A**) Representative images of hematoxylin and eosin (H&E)-stained cells from controls, RA-treated cells or starved cells at 7 days. The graphs show the following findings: (**B**) the maximum and minimum cell body diameters (Dmax and Dmin, respectively); (**C**) the polarization index (Dmax/Dmin); (**D**) the number of cell processes; (**E**) the length of the longest cell process. RA, retinoic acid; St, starvation. Values are given as mean ± S.E.M. from *n* = 100 cells per experimental group obtained in three independent experiments. * *p* < 0.05 compared to controls; ^#^ *p* < 0.05 compared with all other groups. Scale bar = 18 μm.

At 7 days, control cells exhibit morphological features similar to those described at 3 days (Figure 4A). In contrast, both RA-treated cells and starved cells show prominent morphological changes. In fact, cells treated for 7 days with RA show an elongated, spindle-like cell body, where cytosol and nucleus appear homogeneously stained with hematoxylin (Figure 4A); meanwhile, in starved cells, the cell body is irregular in shape, the cytosol is vacuolated, and the nucleus is markedly stained with hematoxylin, suggesting the occurrence of a condensed chromatin (Figure 4A). Quantitative measurements of cell morphology demonstrate that RA-treated cells exhibit the highest Dmax and a lower Dmin compared with controls (Figure 4B), which produces a significant increase in the polarization index (Figure 4C). In contrast, in starved cells, where both the Dmax and Dmin are significantly reduced compared with controls (Figure 4B), the polarization index does not change compared with that of the control cells (Figure 4C). When cell processes were measured, we found that, in RA-treated cells, the reduction in the number of processes per cell and the increase in the length of the longest cell process persist at 7 days (Figure 4D,E, respectively). This is in contrast with 7-day-starved cells, where the number of processes per cell remains unchanged compared with controls (Figure 4D), but the length of the longest cell process is significantly reduced compared with both RA-treated and control cells (Figure 4E). These findings indicate that, at this prolonged time point, RA induces a polarized cell body and a marked elongation of cell process; meanwhile, starvation does not change cell polarization and produces opposite effects on the cell process.

### 2.3. RA Induces Differentiation of SH-SY5Y Cells Towards a Neuron-Like Phenotype

The effects of RA on the differentiation of SH-SY5Y cells were investigated with light microscopy through immunofluorescence for specific antigens, namely the stemness protein nestin and the early and late neuronal markers βIII-tubulin and NeuN, respectively.

**Figure 5:**
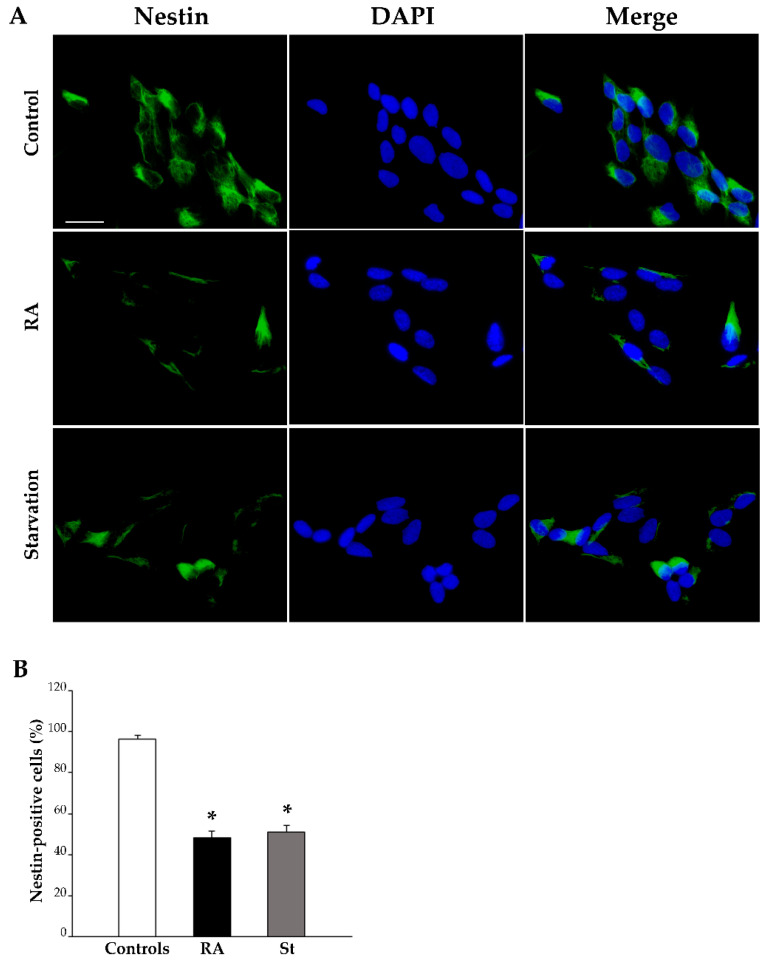
RA and starvation suppress nestin immunofluorescence at 3 days. (**A**) Representative pictures of the immunofluorescence for the stemness marker nestin (green), the fluorescence for the nuclear marker 4′,6-diamidin-2-fenilindolo (DAPI) (blue), and their merge in control cells, RA-treated cells, and starved cells at 3 days. (**B**) The graph reports the count of nestin-positive cells. RA, retinoic acid; St, starvation. Values are given as mean percentage ± S.E.M. from three independent experiments. * *p* < 0.05 compared to controls. Scale bar = 27 μm.

After 3 days of culture in baseline conditions (control), nestin immunofluorescence is widespread; meanwhile, at the same time interval following a single dose of RA, nestin immunofluorescence is suppressed as much as after 3 days of starvation (representative pictures in Figure 5A). The number of nestin-positive cells is indicated the graph in Figure 5B and reported in Table 1. In detail, after 3 days, the number of nestin-positive cells is reduced by half in RA-treated cells and starved cells compared with controls.

**Figure 6:**
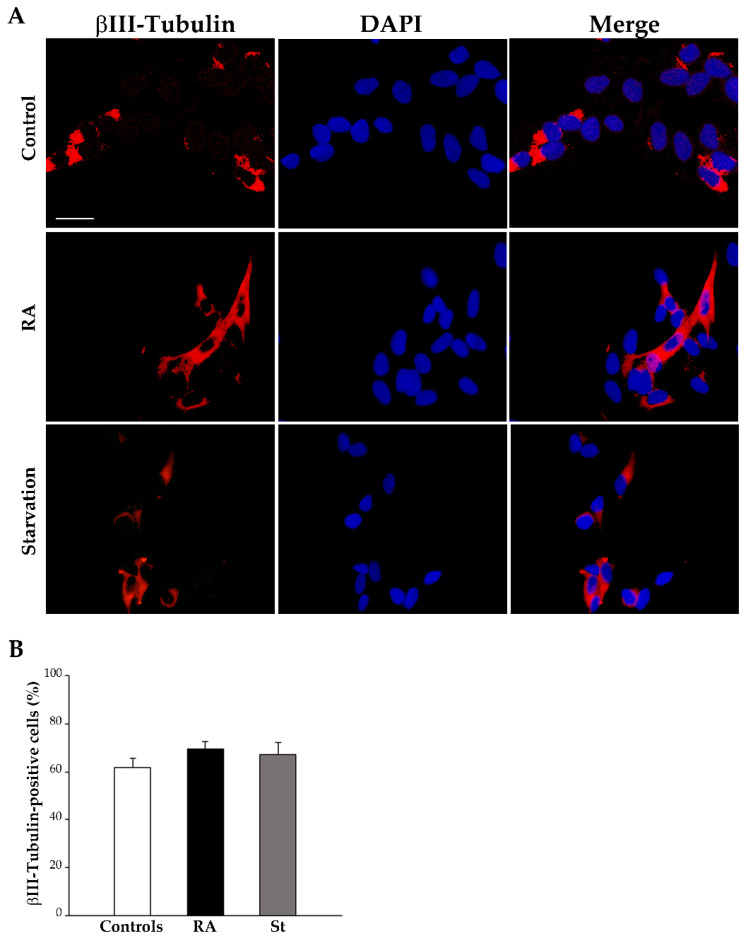
RA and starvation do not change βIII-tubulin immunofluorescence at 3 days. (**A**) Representative pictures of the immunofluorescence for the early neuronal marker βIII-tubulin (red), the fluorescence for the nuclear marker 4′,6-diamidin-2-fenilindolo (DAPI) (blue), and their merge in control cells, RA-treated cells, and starved cells at 3 days. (**B**) The graph reports the count of βIII-tubulin-positive cells. RA, retinoic acid; St, starvation. Values are given as mean percentage ± S.E.M. from three independent experiments. Scale bar = 27 μm.

In contrast, at this time point, βIII-tubulin immunofluorescence appears slightly modified following RA treatment or starvation compared with controls (Figure 6A). This is confirmed by the count of βIII-tubulin-positive cells, which is reported in the graph (Figure 6B) and in Table 1. In detail, these data indicate that the percentage of βIII-tubulin-positive cells slightly, but not significantly, increases either after 3 days of exposure to RA or following 3 days of starvation compared with control cells.

**Figure 7:**
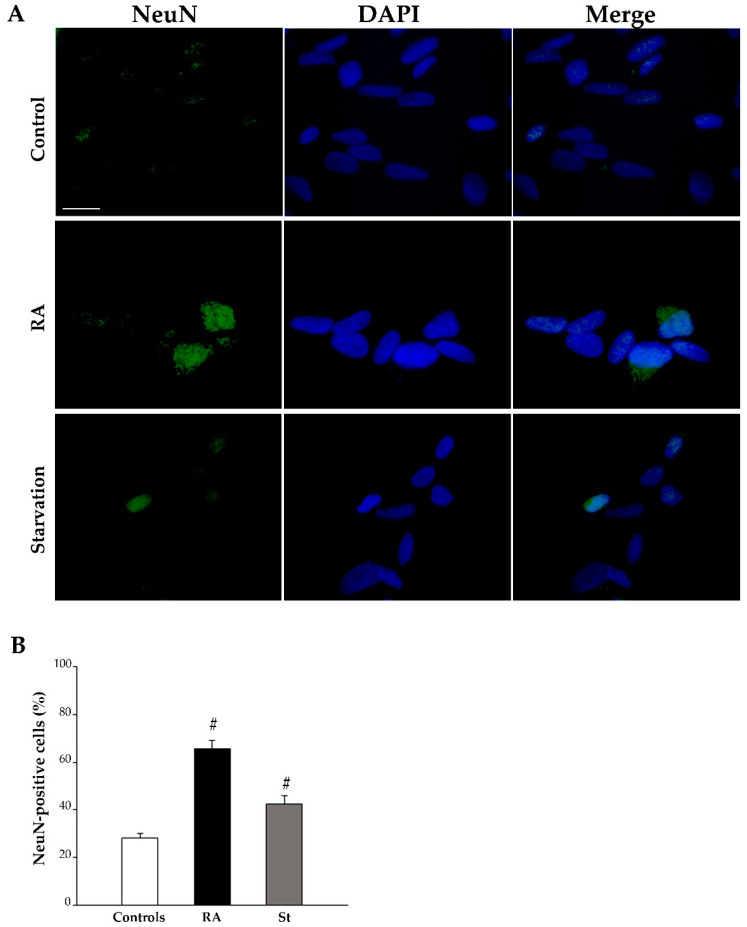
RA and starvation increase NeuN immunofluorescence at 3 days. (**A**) Representative pictures of the immunofluorescence for the late neuronal marker NeuN (green), the fluorescence for the nuclear marker 4′,6-diamidin-2-fenilindolo (DAPI) (blue), and their merge in control cells, RA-treated cells, and starved cells at 3 days. (**B**) The graph reports the count of NeuN-positive cells. RA, retinoic acid; St, starvation. Values are given as mean percentage ± S.E.M. from three independent experiments. ^#^ *p* < 0.05 compared with all other groups. Scale bar = 27 μm.

Finally, at 3 days, NeuN immunofluorescence is rarely observed within the nuclei of control cells, whereas it increases after treatment with either RA or starvation (Figure 7A). The graph in Figure 7B and Table 1 report the count of NeuN-positive cells, which significantly increases in both RA-treated cells and starved cells compared with controls.

**Figure 8:**
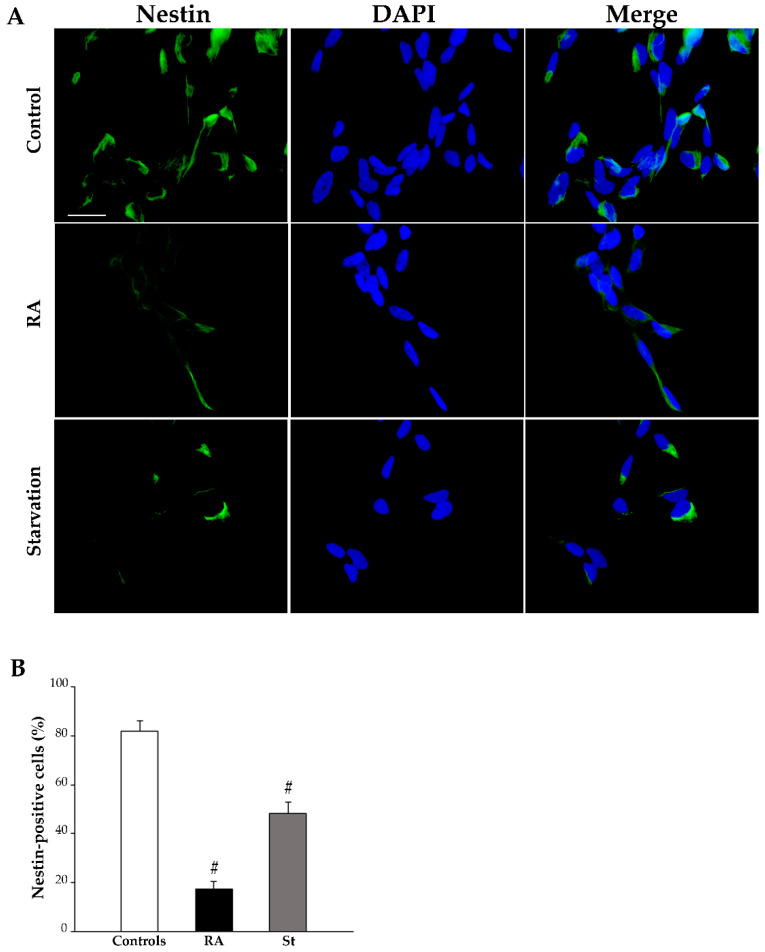
RA markedly reduces nestin immunofluorescence at 7 days. (**A**) Representative pictures of the immunofluorescence for the stemness marker nestin (green), the fluorescence for the nuclear marker 4′,6-diamidin-2-fenilindolo (DAPI) (blue), and their merge in control cells, RA-treated cells, and starved cells at 7 days. (**B**) The graph reports the count of the nestin-positive cells. RA, retinoic acid; St, starvation. Values are given as mean percentage ± S.E.M. from three independent experiments. ^#^ *p* < 0.05 compared with all other groups. Scale bar = 27 μm.

**Figure 9:**
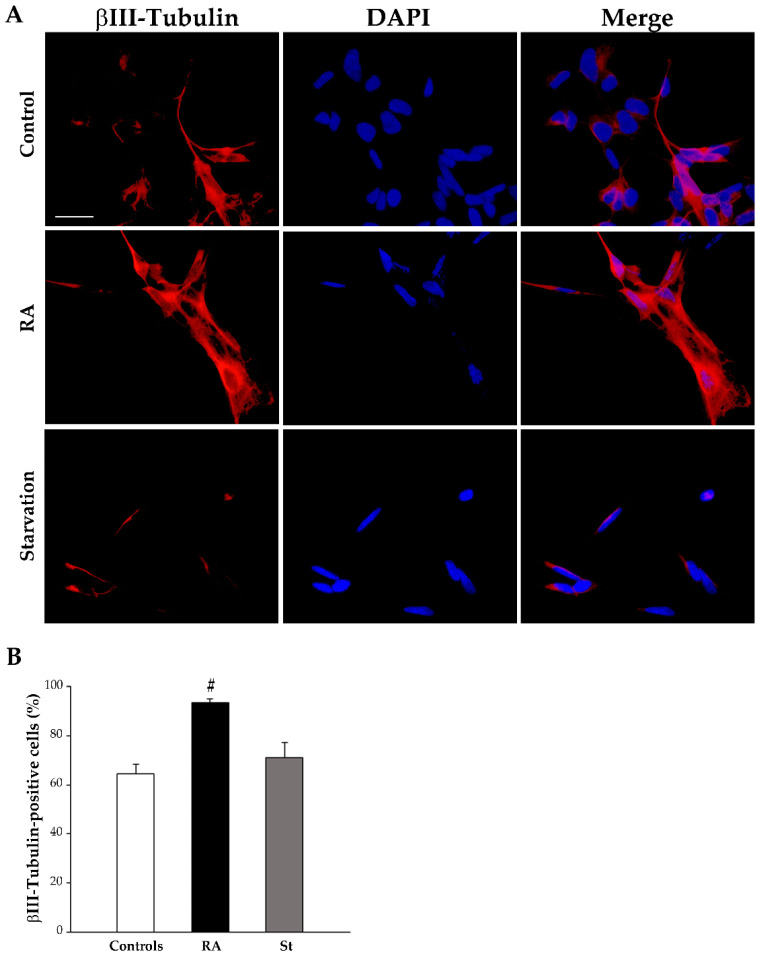
RA markedly increases βIII-tubulin immunofluorescence at 7 days. (**A**) Representative pictures of the immunofluorescence for the early neuronal marker βIII-tubulin (red), the fluorescent nuclear maker 4′,6-diamidin-2-fenilindolo (DAPI) (blue), and their merge in control cells, RA-treated cells, and starved cells at 7 days. (**B**) The graph reports the count of βIII-tubulin-positive cells. RA, retinoic acid; St, starvation. Values are given as mean percentage ± S.E.M. from three independent experiments. ^#^ *p* < 0.05 compared with all other groups. Scale bar = 27 μm.

**Figure 10:**
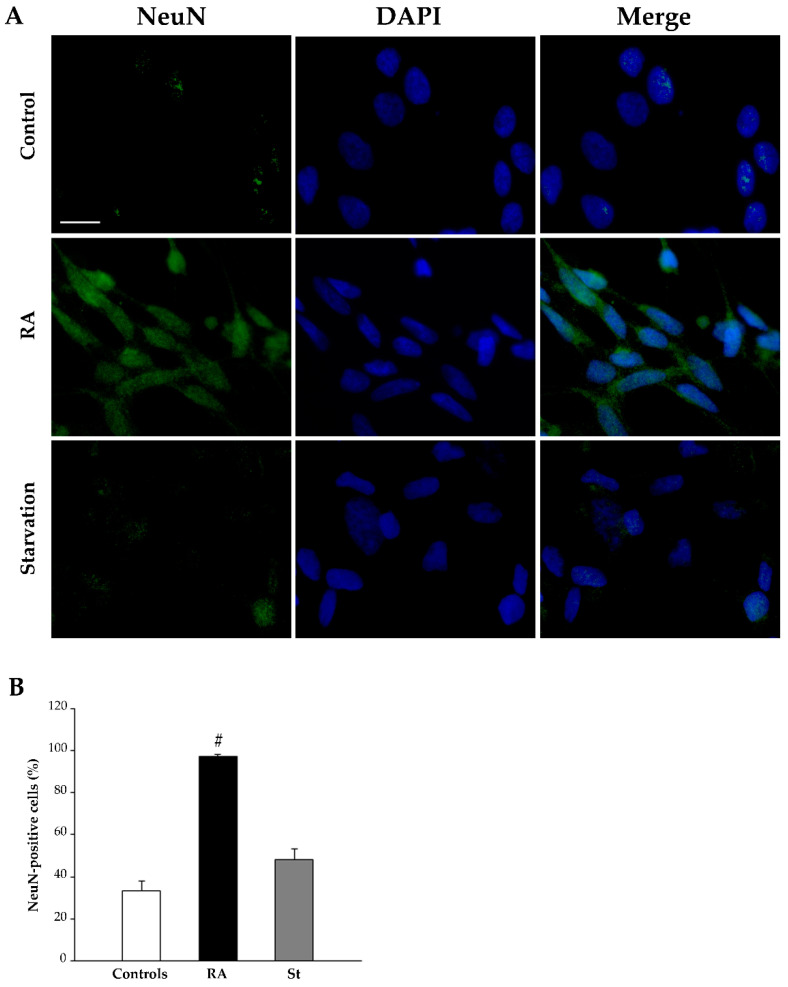
RA markedly increases NeuN immunofluorescence at 7 days. (**A**) Representative pictures of the immunofluorescence for the late neuronal marker NeuN (green), the fluorescent nuclear marker 4′,6-diamidin-2-fenilindolo (DAPI) (blue), and their merge in control cells, RA-treated cells, and starved cells at 7 days. (**B**) The graph reports the count of NeuN-positive cells. RA, retinoic acid; St, starvation. Values are given as mean percentage ± S.E.M. from three independent experiments. ^#^ *p* < 0.05 compared with all other groups. Scale bar = 27 μm.

At 7 days, treatment with RA strongly reduces nestin-immunopositive cells (representative pictures in Figure 8A, graph in Figure 8B, and Table 1), while increasing both βIII-tubulin-positive cells (representative pictures in Figure 9A, graph in Figure 9B, and Table 1) and NeuN-positive cells (representative pictures in Figure 10A, graph in Figure 10B, and Table 1). In contrast, after 7 days of starvation, despite a reduction in nestin, immunofluorescence is still evident in starved cells compared with controls (representative pictures in Figure 8A, graph in Figure 8B, and Table 1), βIII-tubulin (representative pictures in Figure 9A, graph in Figure 9B, and Table 1), and NeuN immunofluorescence (representative pictures in Figure 10A, graph in Figure 10B, and Table 1), are similar to controls.

Table 1 reports the comparison between the percentage of cells, which are immunopositive for each protein at 3 days and at 7 days. These data show that only following exposure to RA is the immunopositivity for these markers measured at 7 days significantly changed compared with that measured at 3 days; this indicates that, at least concerning these phenotypic changes, RA is able to induce a time-dependent differentiation of SH-SY5Y cells that progresses towards a neuron-like phenotype.

### 2.4. RA Increases the Immunofluorescence for the Autophagy Proteins Beclin 1 and LC3 in SH-SY5Y Cells

**Figure 11:**
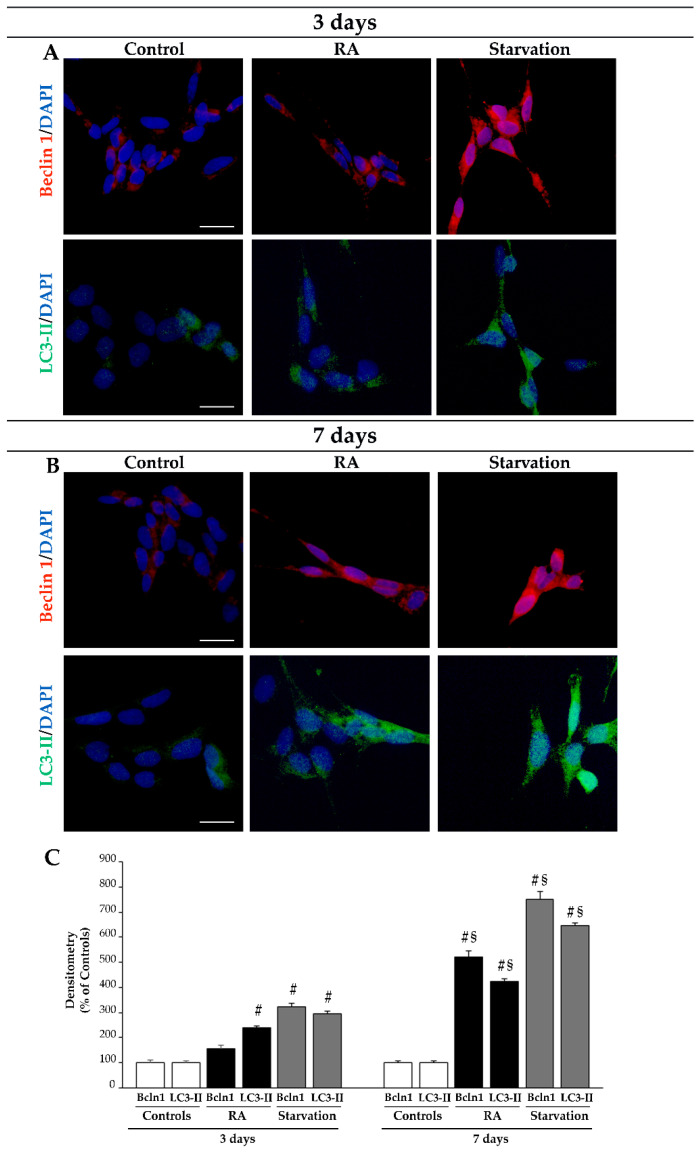
RA increases Beclin 1 and LC3-II immunofluorescence. (**A**,**B**) Representative pictures of the immunofluorescence for the autophagy proteins Beclin 1 (red) and LC3-II (green) in controls and following treatment with RA or starvation at (**A**) 3 days and (**B**) 7 days. (**C**) The graph reports the measure of densitometry. Bcln1, Beclin 1; DAPI, 4′,6-diamidin-2-fenilindolo; LC3-II, microtubule-associated protein 1A/1B-light chain 3 conjugate; RA, retinoic acid. Values are given as mean ± S.E.M. from n = 60 cells per experimental group obtained in three independent experiments. ^#^ *p* < 0.05 compared with all other groups; ^§^ *p* < 0.05 compared with 3 days. Scale bar = 27 μm.

Occurrence of autophagy during neuronal differentiation triggered by RA in SH-SY5Y cells was initially investigated using immunocytochemistry with light microscopy for the autophagy proteins Beclin 1 and microtubule-associated protein 1A/1B-light chain 3 (LC3) (Figure 11). In particular, we used primary antibodies directed against the LC3–phosphatidylethanolamine conjugate (LC3-II), which is the lipidated form of LC3, that is specifically located on the autophagosome membrane [71,72]. As expected, starvation significantly increases the immunofluorescence for both Beclin 1 and LC3-II compared with control conditions and treatment with RA both at 3 days and 7 days (representative pictures in Figure 11A,B, respectively, and graph in Figure 11C). Following 3 days of RA treatment, only the immunofluorescence for LC3-II is increased compared with that of the controls (representative pictures in Figure 11A and graph in Figure 11C), while prolonging RA exposure until 7 days leads to a significant increase in both Beclin 1 and LC3-II immunofluorescence compared with controls (representative pictures in Figure 11B and graph in Figure 11C). Remarkably, at both time intervals, the increase in Beclin 1 and LC3-II immunofluorescence within starved cells is far in excess compared with that found in RA-treated cells (representative pictures in Figure 11A,B, respectively, and graph in Figure 11C). It is notable the occurrence of LC3-II immunofluorescence as “puncta”, which suggests the vacuolar placement of lipidated LC3.

### 2.5. RA Induces Autophagy-like Vacuoles in SH-SY5Y Cells

**Figure 12:**
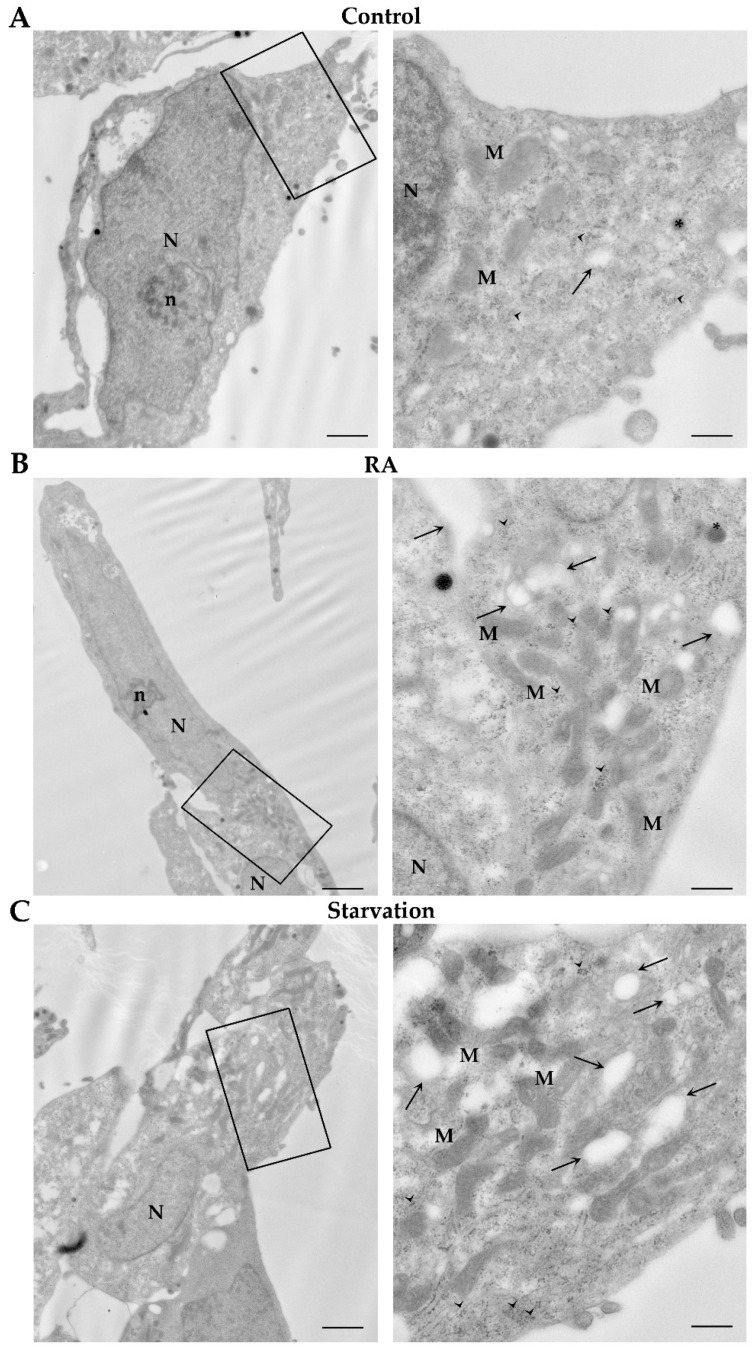
Ultrastructural changes induced by RA and starvation in SH-SY5Y cells at 3 days. Representative micrographs of (**A**) a control cell, (**B**) an RA-treated cell, and (**C**) a starved cell at 3 days. In the right column, the high-magnification pictures correspond to the areas within the frames. Note the abundant glycogen deposits, which form typical rosette-like aggregates. M, mitochondria; n, nucleolus; N, nucleus; RA, retinoic acid. Arrowheads, glycogen; arrows, autophagy-like vacuoles; asterisks, catecholamine granule. Scale bars = (**A**,**C**) 1.2 μm; (**B**) 2 μm; inserts (**A**,**C**) 400 nm; insert (**B**) 500 nm.

**Figure 13:**
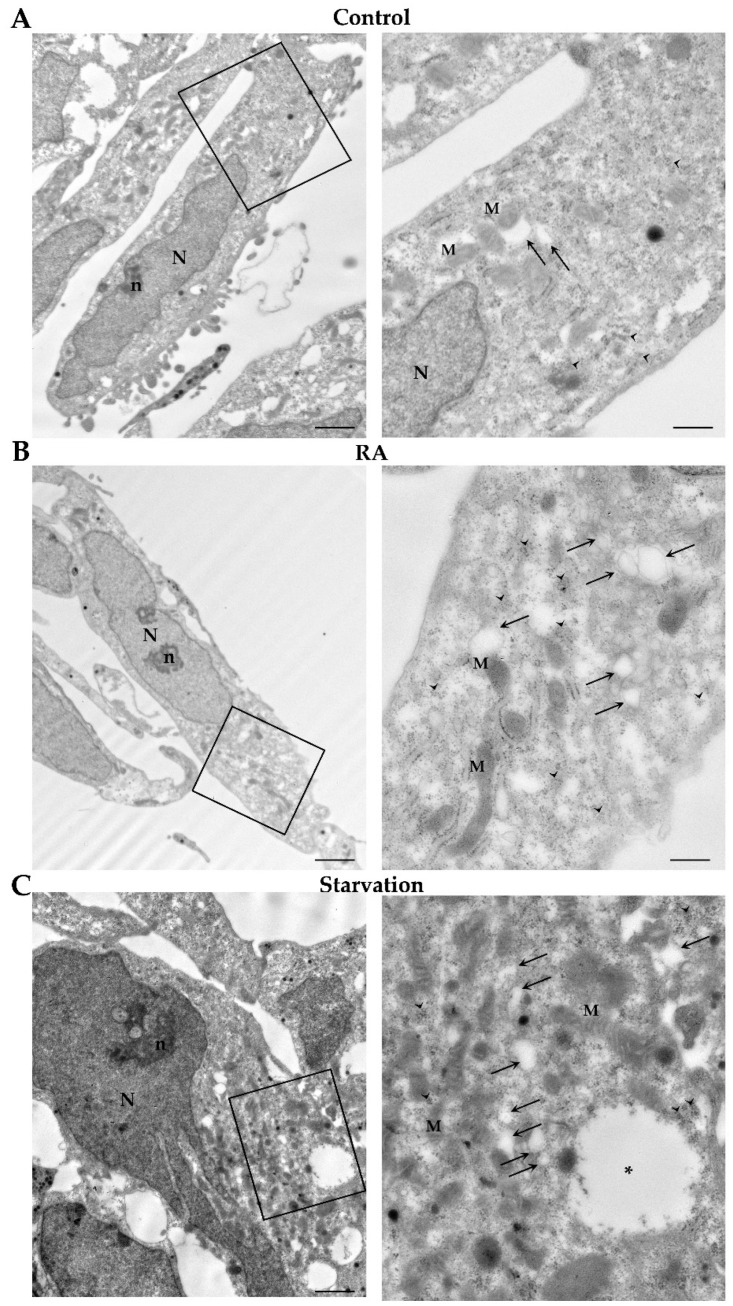
Ultrastructural changes induced by RA and starvation in SH-SY5Y cells at 7 days. Representative micrographs of (**A**) a control cell, (**B**) an RA-treated cell, and (**C**) a starved cell at 7 days. On the right column, the high-magnification pictures correspond to the areas within the frames. Note the presence of a large faint area within a starved cell, where neither organelles nor molecular structures are visible (*). M, mitochondria; n, nucleolus; N, nucleus. Arrowheads, glycogen; arrows, autophagy-like vacuoles. Scale bars = (**A**,**C**) 1.2 μm; (**B**) 1.7 μm; inserts (**A**,**C**) 400 nm; insert (**B**) 470 nm.

The ultrastructure of SH-SY5Y cells grown in baseline conditions (controls), starvation, or in the presence of RA for 3 days or 7 days is shown in Figure 12 and Figure 13, respectively. Specifically, at 3 days, control cells possess a large nucleus surrounded by abundant cytosol, containing widespread glycogen deposits, with a typical rosette-like conformation. Moreover, a few catecholamine granules are visible within the cytosol along with well-conformed mitochondria, several rough endoplasmic reticulum membranes, and rare autophagy-like vacuoles (Figure 12A). Similar ultrastructural features are persistent following 7 days of culture in control conditions (Figure 13A).

In cells treated with RA for 3 days, nuclei show a dispersed chromatin and a well-evident nucleolus. Mitochondria and autophagy-like vacuoles are well evident within the cytosol, where they form perinuclear clusters, intermingled with abundant glycogen deposits (Figure 12B). These ultrastructural features mostly overlap those observed in SH-SY5Y cells grown for 3 days in conditions of starvation (Figure 12C).

At 7 days, in RA-treated cells nuclei appear similar to those described at 3 days. Autophagy-like vacuoles are abundant within the cytosol, where glycogen rosettes are still evident close to perinuclear clusters of mitochondria (Figure 13B). In contrast, following 7 days of starvation, cells exhibit shrunken and irregularly shaped nuclei. Mitochondria and autophagy-like vacuoles are scattered throughout a non-homogeneous cytosol (Figure 13C).

**Figure 14:**
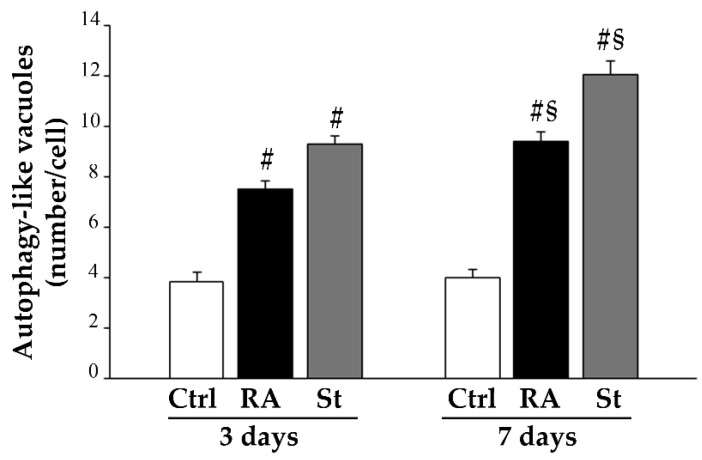
RA induces autophagy-like vacuoles in SH-SY5Y cells. The graph reports the number of autophagy-like vacuoles counted within the cytosol of control cells, RA-treated cells, and starved cells at 3 days and 7 days. Ctrl, controls; RA, retinoic acid; St, starvation. Values are given as the mean ± S.E.M. from *n* = 30 cells per experimental group. ^#^ *p* < 0.05 compared with other groups; ^§^ *p* < 0.05 compared with 3 days.

The count of autophagy-like vacuoles shows a time-dependent increase in these organelles within both RA-treated and starved cells compared with controls. Interestingly, at each time interval, autophagy-like vacuoles counted in starved cells exceed those counted in RA-treated cells (Figure 14).

### 2.6. RA Increases the Key Autophagy Proteins Beclin 1 and LC3 in SH-SY5Y Cells

**Figure 15:**
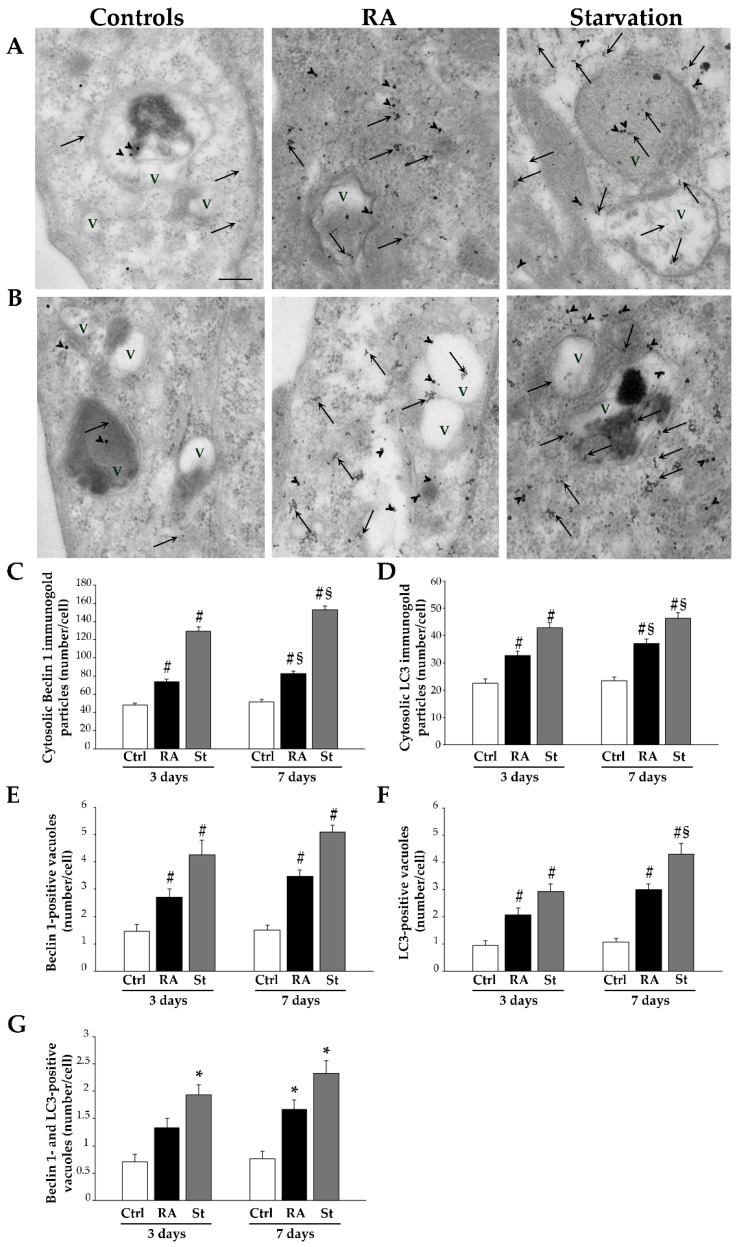
RA increases the autophagy-related proteins Beclin 1 and LC3 in SH-SY5Y cells. (**A**,**B**) Representative micrographs of double immunogold against Beclin 1 (10 nm immunogold particles, arrows) and LC3 (20 nm immunogold particles, arrowheads) in controls and following RA or starvation after 3 days (**A**) or 7 days (**B**). The graphs report the counts of the following: (**C**) cytosolic Beclin 1 immunogold particles; (**D**) cytosolic LC3 immunogold particles, (**E**) Beclin 1-positive vacuoles; (**F**) LC3-positive vacuoles, (**G**) double Beclin 1- and LC3-positive vacuoles. Ctrl, controls; LC3, microtubule-associated protein 1A/1B-light chain 3; RA, retinoic acid; St, starvation; V, autophagy vacuoles. Values are given as the mean ± S.E.M. from *n* = 30 cells per experimental group. * *p* < 0.05 compared with controls; ^#^ *p* < 0.05 compared with other groups; ^§^ *p* < 0.05 compared with 3 days. Scale bar = 216 nm.

Immunogold for the autophagy proteins Beclin 1 and LC3 was carried out to visualize the specific subcellular compartments where these proteins are placed within control cells, RA-treated cells, and starved cells, at 3 days (Figure 15A) and at 7 days (Figure 15B). In control cells, both at 3 days and at 7 days, a few Beclin 1 and LC3 immunogold particles are found scattered in the cytosol, close to vesicles and membranous structures (Figure 15A,B). At 3 days, a number of Beclin 1 and LC3 particles appear in the cytosol of both RA-treated and starved cells, along with vacuoles, which are positive for Beclin 1 and/or LC3 (Figure 15A). At 7 days, such features are persistent and apparently increase in both starved and RA-treated cells (Figure 15B).

The stoichiometric counts of Beclin 1 and LC3 immunogold particles show that, at 3 days, both Beclin 1 (Figure 15C) and LC3 (Figure 15D) are significantly increased in the cytosol of starved and RA-treated cells compared with controls. This increase appears more marked in the starved cells, where both these antigens are more abundant than those counted in RA-treated cells (Figure 15C,D). At this time interval, the number of vacuoles which are positive for Beclin 1 (Figure 15E) or LC3 (Figure 15F) significantly increases both after RA treatment and starvation compared with controls. Again, within starved cells, such an increase is still more pronounced than in RA-treated cells (Figure 15E,F). Moreover, starved cells show a significant increase even in Beclin 1 and LC3 double-stained vacuoles compared with both controls and RA-treated cells (Figure 15G). At 7 days, all these autophagy-related structures further increase within both RA-treated cells and starved cells (Figure 15C–G). Remarkably, at this time interval, the increase in such autophagy-related proteins and organelles is markedly prominent within starved cells.

### 2.7. Autophagy Is Essential for RA-Induced Differentiation of SH-SY5Y Cells

**Figure 16:**
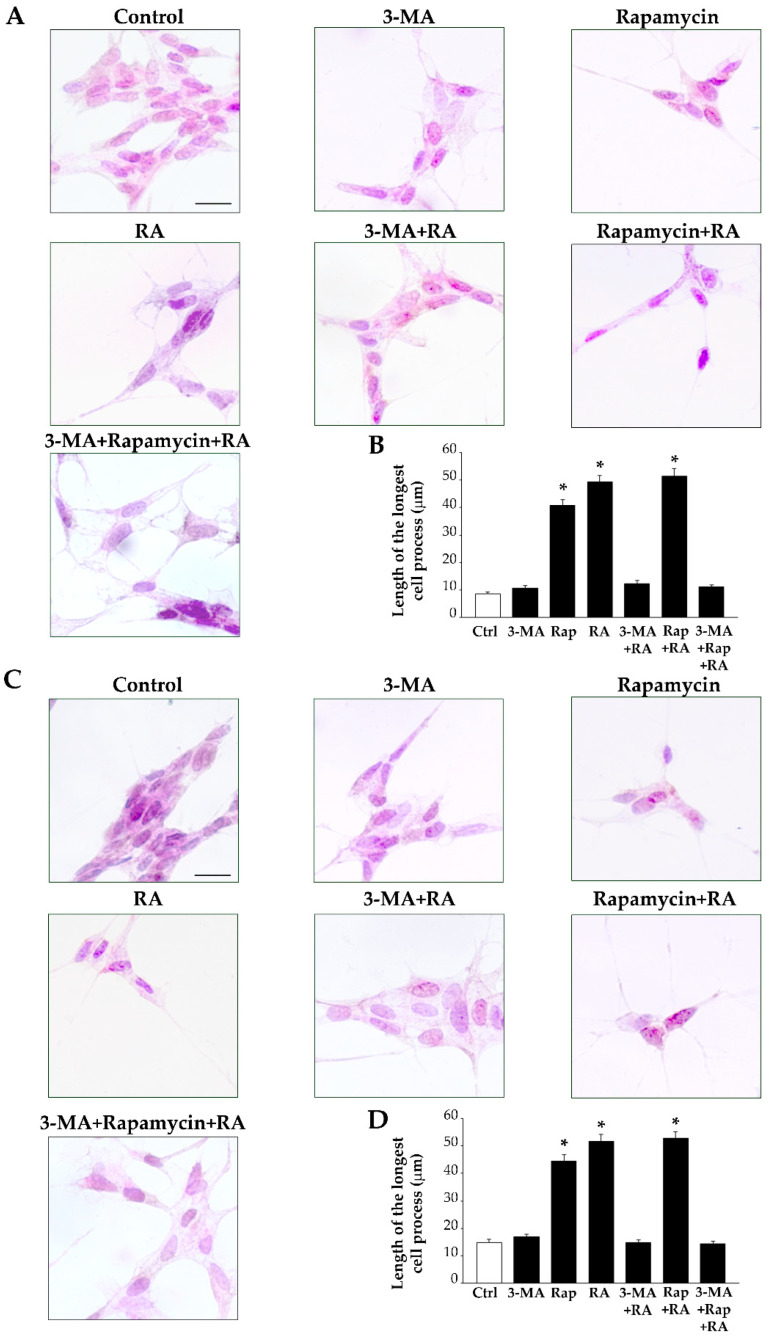
Autophagy is required for morphological changes induced by RA in SH-SY5Y cells. (**A**) Representative images of hematoxylin and eosin (H&E)-stained cells from controls and following treatment with 3-MA, rapamycin, and RA alone or in combination with 3-MA, rapamycin, and 3-MA + rapamycin at 3 days. (**B**) The graph reports the length of the longest cell process. (**C**) Representative images of H&E-stained cells from controls and following treatment with 3-MA, rapamycin, and RA alone or in combination with 3-MA, rapamycin, and 3-MA + rapamycin at 7 days. (**D**) The graph reports the length of the longest cell process. 3-MA, 3-methyladenine; Ctrl, controls; RA, retinoic acid; Rap, rapamycin. Values are given as mean ± S.E.M. from *n* = 50 cells per experimental group obtained in three independent experiments. * *p* < 0.05 compared with controls. Scale bars = 29 μm.

In order to ascertain whether the activation of autophagy is essential during RA-induced cell differentiation, we treated SH-SY5Y cells with the autophagy inhibitor 3-MA or the autophagy stimulator rapamycin and we analyzed the effects of such autophagy modulators on cell differentiation produced by RA. H&E-stained SH-SY5Y cells treated with 3-MA for 3 days appear similar to control cells (Figure 16A); meanwhile, cells treated with rapamycin exhibit a fusiform cell body and a few long cell processes, which are reminiscent of the typical cell morphology produced by treatment with RA (Figure 16A). Interestingly, in cells treated with 3-MA + RA, the inhibition of autophagy mostly occludes the RA-induced morphological changes (Figure 16A), which conversely are enhanced when the autophagy stimulator rapamycin is administered in combination with RA (Figure 16A). Measurement of the length of the cell processes, which is powerfully increased by RA, indicating the acquisition of a neuronal-like phenotype [73], shows that inhibition of autophagy by 3-MA prevents the RA-induced cell process elongation, while rapamycin by itself and in combination with RA significantly increases the length of the cell processes (Figure 16B). After 7 days of treatment, the effects of 3-MA and rapamycin on RA-induced neuronal-like differentiation described above appear even more evident (Figure 16C,D).

**Figure 17:**
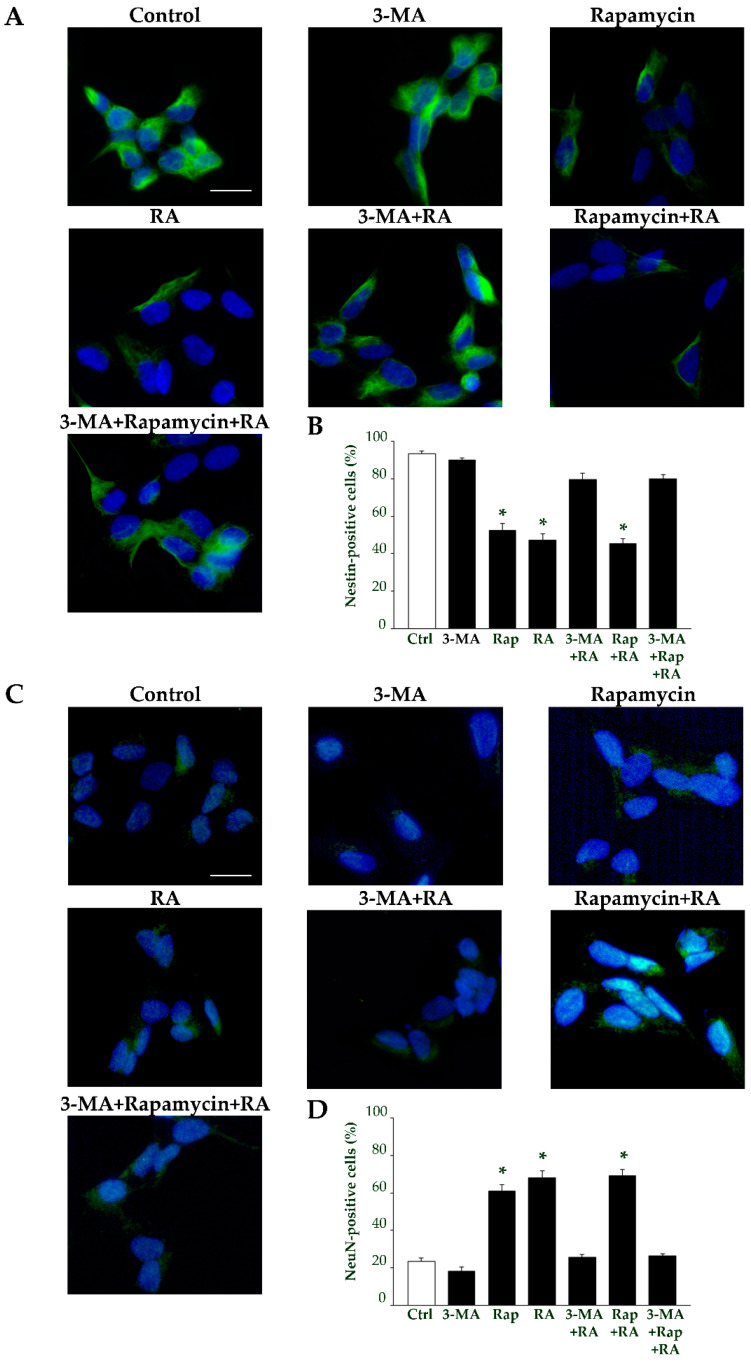
Autophagy modulates the effects of RA on nestin and NeuN immunofluorescence at 3 days. (**A**) Representative pictures of nestin immunofluorescent cells (green) merged with the fluorescent nuclear marker 4′,6-diamidin-2-fenilindolo (DAPI) (blue), from controls and following treatment with 3-MA, rapamycin, and RA alone or in combination with 3-MA, rapamycin, and 3-MA + rapamycin. (**B**) The graph reports the count of the nestin-positive cells. (**C**) Representative pictures of NeuN immunofluorescent cells (green) merged with the fluorescent nuclear DAPI (blue), from controls and following treatment with 3-MA, rapamycin, and RA alone or in combination with 3-MA, rapamycin, and 3-MA + rapamycin. (**D**) The graph reports the count of the NeuN-positive cells. 3-MA, 3-methyladenine; Ctrl, controls; RA, retinoic acid; Rap, rapamycin. Values are given as mean percentage ± S.E.M. from three independent experiments. * *p* < 0.05 compared to controls. Scale bars = 30 μm.

The effects of autophagy modulation on the immunopositivity for specific markers were also investigated. Specifically, immunofluorescence for nestin and NeuN following 3 days of treatment with RA in the presence of 3-MA or rapamycin is shown in Figure 17. Briefly, immunofluorescence for nestin appears toned down in the presence of rapamycin, while it is widespread in the presence of 3-MA (Figure 17A). As reported in the graph (Figure 17B), the percentage of nestin immunofluorescent cells is decreased in rapamycin-treated cells compared with controls, while it is similar to controls in 3-MA-treated cells. Remarkably, a similar trend is measured when the autophagy stimulator or the autophagy inhibitor are administered in combination with RA (Figure 17B).

Conversely, as shown in the representative Figure 17C, immunofluorescence for NeuN appears very scattered in the presence of 3-MA, while it is widespread in the presence of rapamycin. In the graph in Figure 17D the percentage of NeuN-positive cells is reported. These data show that treatments which stimulate autophagy produce a significant increase in the amount of NeuN-immunofluorescent cells compared with controls (Figure 17D). In contrast, after treatments which inhibit autophagy the amount of NeuN-immunofluorescent cells remains similar to that of controls (Figure 17D).

**Figure 18:**
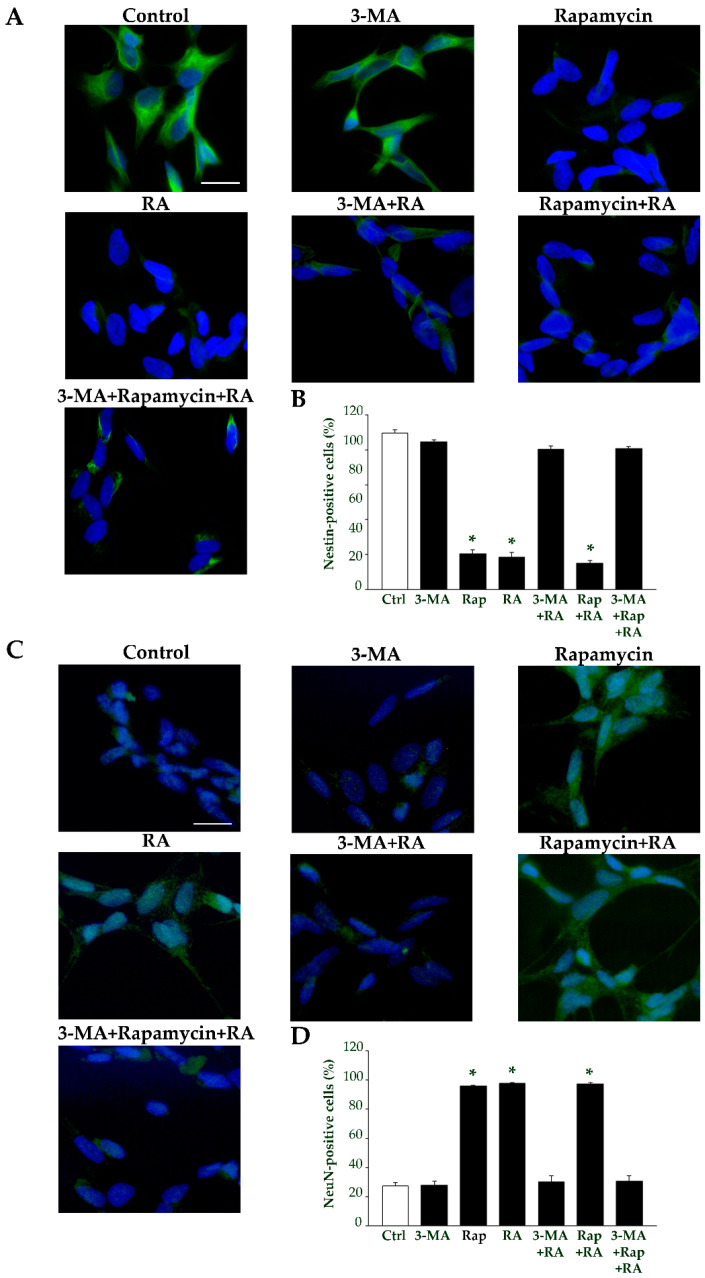
Autophagy modulates the effects of RA on nestin and NeuN immunofluorescence at 7 days. (**A**) Representative pictures of nestin immunofluorescent cells (green) merged with the fluorescent nuclear marker 4′,6-diamidin-2-fenilindolo (DAPI) (blue), from controls and following treatment with 3-MA, rapamycin, and RA alone or in combination with 3-MA, rapamycin, and 3-MA + rapamycin. (**B**) The graph reports the count of the nestin-positive cells. (**C**) Representative pictures of NeuN immunofluorescent cells (green) merged with the fluorescent nuclear marker DAPI (blue), from controls and following treatment with 3-MA, rapamycin, and RA alone or in combination with 3-MA, rapamycin, and 3-MA + rapamycin. (**D**) The graph reports the count of the NeuN-positive cells. 3-MA, 3-methyladenine; Ctrl, controls; RA, retinoic acid; Rap, rapamycin. Values are given as mean percentage ± S.E.M. from three independent experiments. * *p* < 0.05 compared to controls. Scale bars = 30 μm.

At 7 days, immunofluorescence for nestin and NeuN shows a very similar trend (Figure 18). These data indicate that the expression of specific differentiation markers induced by RA is strictly dependent on the presence of a concomitant ongoing autophagy.

## 3. Discussion

In the present study, we demonstrated that RA-induced differentiation of SH-SY5Y cells is associated with an increase in the key autophagy proteins Beclin 1 and LC3.

Such an RA-induced increase in Beclin 1 and LC3 was documented under both light immunofluorescence and immunoelectron microscopy. Specifically, the increase in LC3-II immunofluorescence following RA witnesses the increase in autophagosomes which represent the hallmark organelles of an activated autophagy [66]. This was further confirmed through the stoichiometry of each protein in situ and within autophagy vacuoles, as demonstrated by TEM. Our findings demonstrate that the effects of RA on cell differentiation are concomitant with an increase in authentic autophagosomes, which suggests that autophagy activation may be involved in RA-induced SH-SY5Y cell differentiation.

Specifically, RA at the single dose of 10 μM produced the following findings: (i) reduction in cell proliferation; (ii) increase in cell polarization; (iv) decrease in nestin immunostaining; (v) increase in βIII-tubulin immunostaining; (vi) increase in NeuN immunostaining. These effects indicate RA-induced cell differentiation and they were found to be concomitant with an increase in key autophagy proteins and autophagy vacuoles.

It is remarkable that both differentiation-related phenomena and autophagy-related hallmarks were evident already at 3 days and persisted at 7 days following a single dose of RA.

Similar effects were produced by starvation in SH-SY5Y cells. Specifically, we found that, in starved cells, the decrease in Ki67-positive cells and the occurrence of a differentiated cell phenotype were concomitant with the increase in autophagy-related structures. In fact, starved cells promptly respond to fasting by halting cell proliferation and activating autophagy [27,28,29,30,31,74,75,76,77], which in turn stimulates neuronal differentiation [44,70,78,79].

These effects were mainly evident after 3 days of starvation. When starvation was prolonged for 7 days, cells show structural and ultrastructural changes, which alter cell morphology and lose the neuronal-like phenotype, which appears at 3 days. Moreover, ultrastructural morphology showed that these alterations occurred in cells possessing the highest amounts of autophagy-related vacuoles and key autophagy proteins.

It is likely that a small autophagy activation, similar to that produced by a single dose of RA, is effective at inducing cell differentiation, which persists at 7 days without impairing cell integrity. The detrimental effects of starvation observed at 7 days in differentiated SH-SY5Y cells are likely to be the consequence of an excess of activation of the autophagy pathway, since starvation was constantly present during the 7-day time interval. Indeed, prolonged autophagy activity may deprive cells of essential energy sources necessary for their survival [31,69,70]. In this way, the differentiation process is impaired by the loss of cell integrity. In order to better explore such an issue, repeated administration of RA could be carried out. Again, higher doses of RA could be challenged. Nonetheless, this is not the purpose of the present study, which aims to analyze the variations in autophagy proteins and organelles following a dose of RA, which is routinely applied to induce cell differentiation.

In this respect, these data demonstrate that RA-induced autophagy is closely associated with RA-induced cell differentiation. In fact, when RA was administered in the presence of autophagy inhibition, cell differentiation is occluded. This suggests that these RA-induced effects are ineffective in inducing cell differentiation if autophagy is suppressed. Witnessing for a role of RA as a powerful autophagy stimulator, the concomitant administration of rapamycin to induce autophagy does not produce a significant effect.

Phenotypical changes induced by RA in SH-SY5Y cells are widely documented. They include the enhancement of neurites outgrowth [80,81,82,83,84] and expression of neuronal markers [22,83,85,86,87,88]. These effects are typically attributed to the RA-induced regulation of a variety of genes involved in neuronal development [18,89,90,91]. Anyway, it is worth pointing out that, in most of these studies, the treatment protocol with RA involved the use of growth media with different composition [2], different fetal bovine serum (FBS) percentages [83,87], different glucose concentrations [88], or combined treatments with other differentiating agents, such as growth factors [22,92]. In our protocol, RA was administered alone to the growth medium, which, apart from the addition of RA, was identical to that used for control cells. Therefore, the effects reported here can be uniquely attributed to RA.

The peculiarity of the effects induced by RA in this study may be due to the slight dose and the short time of exposure (single dose) compared with the drastic and continuous stimulation achieved following 7 days of starvation.

The most striking morphological effects induced by RA were the marked elongation of a few (often only one or two) cell processes and the increase in cell polarization. These features can be considered to be closely related to a differentiated cell phenotype, according to Cowley and coll. [73]. In this respect, on the basis of such morphological data, we cannot establish whether cell processes induced by RA are authentic neurites. This intriguing hypothesis should be challenged only through electrophysiological studies, which may contribute to further interpretation of these morphological findings. Here, we describe only morphological variations, which are consistent with those of general cell differentiation.

These RA-induced phenotypical changes were accompanied by an increase in autophagy-related structures, which remained always lower compared with those produced by starvation, suggesting that, in cells treated with a single dose of 10 μM RA, autophagy is slightly activated compared with starved cells. In line with this, ultrastructure and stoichiometry demonstrate that the increase in autophagy-related structures measured in starved cells at 7 days was dramatically higher compared with that measured at 3 days following starvation and at 3 days and 7 days following a single dose of RA.

Finally, the preservation of cell integrity following RA-induced autophagy activation may be explained by an alternative mechanism, which involves the marked protective effect of Bcl-2. In fact, RA is a strong inducer of Bcl-2 [93,94,95], whose overexpression prevents cell death following an excess of autophagy produced by prolonged starvation [96].

## 4. Materials and Methods

### 4.1. Cell Cultures

SH-SY5Y cells line, obtained from the American Type Culture Collection (ATCC, Rockville, MD, USA), was grown in Ham’s F12 nutrient medium containing 2 mM glutamine (Sigma-Aldrich, St. Louis, MO, USA) and supplemented with 10% heat-inactivated fetal bovine serum (FBS) and 1% penicillin/streptomycin (Sigma-Aldrich). Cells were kept in a wet atmosphere with 5% CO_2_ at 37 °C. Experiments were performed when cells were in the log-phase of growth and at 70% confluence.

Twenty-four hours before the treatments, cells were seeded at different densities, depending on the specific analysis. For cytochemistry and immunocytochemistry with light microscopy, 4 × 10^4^ SH-SY5Y cells were seeded on cover slips, which were placed within 24-well plates, with a final volume of 1 mL/well.

For TEM, 1 × 10^6^ SH-SY5Y cells were seeded in 6-well plates, with a final volume of 2 mL/well.

Following seeding, cells were incubated at 37 °C in 5% CO_2_ for 24 h and then they were treated with RA or were grown under starvation conditions.

### 4.2. Cell Treatments and Experimental Design

SH-SY5Y cells were treated with the differentiating agent RA, with a single dose of 10 µM, which was selected since it is known to induce cell differentiation [2,97]. In detail, a stock solution of 1 mM RA (Sigma-Aldrich) was prepared by dissolving 0.3 mg of RA powder in 1 mL of 100% ethanol. Appropriate aliquots of RA stock solution were diluted within the cell culture medium to obtain the final treatment solution of 10 µM RA in 1% ethanol.

Pilot studies carried out to disclose potential effects of 1% ethanol within the basic medium revealed no differences between cells grown in the medium added with 1% ethanol and cells grown in the basic medium. Therefore, control cells were exposed to the basic medium.

To produce starvation, a condition in which autophagy is physiologically activated as a mechanism of cell survival [26], SH-SY5Y cells were grown in a starvation medium comprising Ham’s F12 supplemented with 0.25% heat-inactivated FBS (Sigma-Aldrich) and 1% penicillin/streptomycin (Sigma-Aldrich), and devoid of amino acids.

The involvement of autophagy in cell differentiation induced by RA was investigated using the autophagy inhibitor 3-MA (Sigma-Aldrich) [98] and the autophagy stimulator rapamycin (Sigma-Aldrich) [99]. In these experiments, cells were exposed to 10 mM 3-MA or 100 nM rapamycin, alone or in combination with 10 μM RA, for 3 days or 7 days. When such autophagy modulators were administered in combination with RA, they were added to the culture medium 2 h before RA. The treatment doses of both 3-MA and rapamycin were selected based on previous studies [100,101,102]. Treatment doses, obtained by a stock solution of 100 mM 3-MA or 1 mM rapamycin, were diluted in the culture medium containing 10% dimethyl sulfoxide (DMSO), which was demonstrated to not affect cell viability [103,104].

Cells were maintained within the specific (treatment or control) medium for 3 days or 7 days; at this point, cell viability, structural, and ultrastructural morphology and expression of proteins related to differentiation or autophagy pathway were assessed using light microscopy. In detail, potential changes in cell proliferation were indirectly inferred using Ki67 immunopositivity, which labels the cell-cycle-associated proliferating protein Ki67 [105,106]. The immunopositivity of the following proteins, which are related to cell differentiation, was also investigated: nestin, as stem-like marker [107]; βIII-tubulin, as an early (immature) neuronal marker [108,109]; NeuN, as a late neuronal marker, which specifically labels post-mitotic neurons [110]. Moreover, the key autophagy proteins Beclin 1 and LC3 were analyzed through immunofluorescence. Beclin 1 is a component of the autophagy initiation complex, and it is located on the pre-vesicular membranous structures which form phagophores and omegasomes [111,112,113]. In the following step, the autophagosome marker LC3 was recruited to the autophagosomal membranes, where it became lipidated and formed LC3-II [71,72,114].

Finally, these very same autophagy proteins and autophagy-like vacuoles were analyzed using TEM, which enables quantitative assessments to be made by utilizing in situ ultrastructural imaging and the stoichiometric immunogold procedure.

### 4.3. Trypan Blue (TB) Staining and Cell Count

At the end of the treatments, SH-SY5Y cells were collected and centrifuged at 800× *g* for 5 min. The cell pellet was suspended in the culture medium and 25 µL of the cell suspension were added to a 62.5 μL solution containing 1% TB (Sigma-Aldrich) and 37.5 μL phosphate-buffered solution (PBS) for 5 min at room temperature (RT). Ten µL of this solution was used to carry out the cell count within a Bürker glass chamber under an Olympus CKX 41 inverted microscope (Olympus Corporation, Tokyo, Japan). Three independent counts of both TB-positive cells and total cells were carried out. The data refer to three independent experiments and are given as the mean ± S.E.M. percentage of TB-positive cells out of the total cells.

### 4.4. Hematoxylin and Eosin (H&E) Cytochemistry, Cell Count, and Cell Morphometry

Cells were fixed with 4% paraformaldehyde in PBS for 15 min at RT, washed with PBS, and then stained for a few minutes with hematoxylin solution (Sigma-Aldrich). Hematoxylin staining was stopped by washing in running water. Then, cells were stained with eosin solution for a few seconds (Sigma-Aldrich). Repeated washing with distilled water was carried out to remove the dye in excess. Cells were dehydrated in increasing alcohol solutions, gently transferred on a slide, clarified in xylene, and covered with dibutylphthalate polystyrene xylene (DPX) mounting medium (Sigma-Aldrich). H&E-stained cells were observed under the Nikon Eclipse Ni light microscope (Nikon, Tokyo, Japan), equipped with a digital camera connected to the NIS Elements software (NIS-Elements D 5.30.00-build 1531-64-bit) for image analysis (Nikon, Tokyo, Japan).

To assess the cell number, H&E-stained cells were counted under 20× magnification within three microscopic fields, where only distinct cells that were not overlapping could be detected.

Values are given as the mean percentage ± S.E.M. (assuming controls as 100%) of counts carried out in three independent experiments.

H&E-stained cells were also used to measure morphological changes induced by the treatments (i.e., Dmax and Dmin of the cell body, number of processes per cell, and length of the longest cell process). In detail, cells were analyzed under the light microscope Nikon Eclipse Ni light microscope (Nikon) at 40× magnification and measures were carried out by using the free software Image J (National Institutes of Health, NIH, Bethesda, MD, USA, Version 1.8.0_172). The cell polarization index was obtained by calculating the Dmax/Dmin. These measures refer to *n* = 100 cells per experimental group. In experiments where autophagy was modulated using 3-MA or rapamycin, the measurement of the cell process was carried out with n = 50 cells per experimental group. Values are given as the mean ± S.E.M. from three independent experiments.

### 4.5. Immunofluorescence

Cell proliferation was analyzed through the mouse monoclonal anti-Ki67 primary antibodies (Ab-I) (Millipore, Billerica, Burlington, MA, USA). To investigate the effects on cell differentiation, the following Ab-I-based approaches were used: rabbit polyclonal anti-nestin Ab-I (Abcam, Cambridge, UK); mouse monoclonal anti-βIII-tubulin Ab-I (Abcam); mouse monoclonal anti-NeuN Ab-I (Millipore). Finally, to investigate changes in the specific autophagy-related proteins Beclin 1 and LC3, the mouse anti-Beclin 1 Ab-I (Abcam), the rabbit anti-LC3 Ab-I (Abcam), and the rabbit LC3-II Ab-I (Abcam) were used.

SH-SY5Y cells were first permeabilized by TritonX 0.1% for 15 min in PBS and then incubated in a blocking solution containing 10% normal goat serum (NGS) in PBS for 1 h at RT. Then, cells were incubated with the Ab-I solution containing 2% NGS in PBS, where the Ab-I were diluted as follows: anti-Ki67 Ab-I (1:100), anti-nestin Ab-I (1:200), anti-βIII-tubulin Ab-I (1:200), anti-NeuN Ab-I (1:100), anti-Beclin 1 Ab-I (1:100), anti-LC3 Ab-I (1:100), and anti-LC3-II Ab-I (1:50). Cells were kept in the Ab-I solution overnight at 4 °C. At the end of the incubation, cells were washed out with PBS and the reactions with the Ab-I were revealed using the appropriate anti-mouse or anti-rabbit fluorescent secondary antibody (Ab-II), Alexa Fluor 488 (1:200, Life Technologies, Carlsbad, CA, USA) or Alexa Fluor 546 (1:200, Life Technologies), for 1 h and 30 min at RT. To visualize the cell nuclei, cells were incubated for 5 min with the nuclear dye 4′,6-diamidin-2-fenilindolo (DAPI) (Sigma-Aldrich) diluted to 1:1000 in distillated water. All these reactions were carried out within the well plates. After washing in PBS, the cover slips were gently pulled out, transferred on a slide, and mounted with the mounting medium, Fluoroshield (Sigma-Aldrich).

The cells were observed using the Nikon Eclipse Ni light microscope equipped with a fluorescent lamp and a digital camera connected to the NIS Elements software (NIS-Elements D 5.30.00-build 1531-64-bit) for image analysis (Nikon, Tokyo, Japan). All experiments were carried out in triplicate.

For Ki67, nestin, βIII-tubulin, and NeuN immunofluorescence, the percentages of positive cells out of total cells were counted under the fluorescent microscope at 20× magnification within three microscopic fields, where only distinct cells that were not overlapping could be detected.

Values are given as the mean percentage ± S.E.M. of positive cells out of total cells, which were counted in three independent experiments.

The densitometry of Beclin 1 and LC3-II immunofluorescence was measured using the software Image J (NIH, Bethesda, MD, USA, Version 1.8.0_172). In detail, the corrected total cell fluorescence was quantified according to Marwaha and Sharma 2017 [115], and values are given as the mean percentage ± S.E.M. of the optical density (assuming controls as 100%) measured in *n* = 60 cells per experimental group. The data refer to the three independent experiments.

### 4.6. Transmission Electron Microscopy

After removing the culture medium, SH-SY5Y cells were fixed in 2.0% paraformaldehyde and 0.1% glutaraldehyde in 0.1 M PBS, pH 7.4, for 1 h and 30 min at 4 °C. Cells were gently scraped from the plate and centrifuged at 10,000 rpm for 10 min to obtain the cell pellet. After they were washed with PBS, cells were post-fixed in 1% osmium tetroxide (OsO_4_) for 1 h at 4 °C. These fixing and post-fixing solutions were validated in previous studies for immunogold ultrastructural morphometry in order to minimally cover the antigen epitopes while preserving tissue architecture fairly well [102,116,117]. Then, the cells were dehydrated in increasing ethanol solutions and finally embedded in epoxy resin.

Ultrathin sections, obtained by cutting samples at the RMC ultramicrotome PowerTome X (Boeckler Instruments, Inc., Tucson, AZ, USA), were stained with uranyl acetate and lead citrate and they were finally examined using the JEOL JEM-100SX TEM (JEOL, Tokyo, Japan).

### 4.7. Post-Embedding Immunocytochemistry and Ultrastructural Morphometry

Ultrathin sections were collected on nickel grids and were incubated on droplets of aqueous sodium metaperiodate for 30 min at RT to remove OsO_4_.

After washing in PBS, grids were incubated in a blocking solution containing 10% NGS and 0.2% saponin for 20 min at RT. Then, grids were incubated with the Ab-I solutions within a humidified chamber overnight, at 4 °C. In detail, these Ab-I solutions contained mouse anti-Beclin 1 Ab-I (Abcam, diluted 1:80), rabbit anti-LC3 Ab-I (Abcam, diluted 1:50), 0.2% saponin, and 1% NGS in PBS. After washing in PBS, grids were incubated with the Ab-II solution, containing the Ab-II conjugated with gold particles, 0.2% saponin, and 1% NGS in PBS for 1 h at RT. In detail, Ab-II against Ab-I anti-LC3 possessed a gold particle diameter of 20 nm (BB International, Cardiff, UK) and was diluted to 1:50, while Ab-II against Ab-I anti-Beclin 1 possessed a gold particle diameter of 10 nm (BB International, Cardiff, UK) and was diluted to 1:80. Negative control sections were incubated with Ab-II only.

Profiting from the ultrastructural imaging and immunogold-based stoichiometry, we measured the numbers of autophagy-related vacuoles and autophagy proteins Beclin-1 and LC3 during RA treatment or starvation.

To this purpose, grids were analyzed using TEM under 8000× magnification, which represents the minimal magnification at which immunogold particles and autophagy-like vacuoles, identified according to Klionsky et al. [114], can be concomitantly visualized [118].

In these sections, according to previous studies [102,119], we counted the following:

(i) Number of autophagy-like vacuoles.
(ii) Number of anti-Beclin 1 and anti-LC3 immunogold particles placed within the cytosol.
(iii) Number of Beclin 1- or LC3-positive vacuoles.
(iv) Number of Beclin 1 and LC3 double-positive vacuoles.

Values are given as the mean ± S.E.M. per cell from *n* = 30 cells per experimental group.

### 4.8. Statistical Analysis

All data were compared by using one-way analysis of variance, ANOVA, followed by Scheffè’s post hoc analysis. The differences between the groups were considered to be significant when the null hypothesis (H_0_) was *p* < 0.05.

## 5. Conclusions

The present study provides morphology-based evidence that RA induces neuronal differentiation of SH-SY5Y cells by increasing specific autophagy proteins and autophagy-related structures. These RA-induced effects may help to preserve cell integrity and support the metabolic changes involved in RA-induced differentiation and cell remodeling.

Remarkably, the suppression of the RA-induced changes on cell morphology and specific differentiation markers, which occurs when autophagy is inhibited, indicates that autophagy plays an essential role in sustaining such effects of RA on SH-SY5Y cells.

On the other hand, the molecular mechanisms underlying the effects of RA on autophagy pathway remain to be elucidated; they are not the focus of the present study. These experiments could be the aim of a future study.

## Figures and Tables

**Table 1:** Percentage of nestin-, βIII-tubulin-, and NeuN-positive cells following 3 days or 7 days of retinoic acid (RA) exposure or starvation.

	Nestin-Positive Cells (%)		βIII-Tubulin-Positive Cells (%)		NeuN-Positive Cells (%)	
	3 Days	7 Days	3 Days	7 Days	3 Days	7 Days
Controls	96.3 ± 1.9	82.0 ± 4.1	61.6 ± 4.2	64.5 ± 4.0	28.1 ± 2.1	33.5 ± 4.5
	*p* = 0.9751		*p* > 0.9999		*p* > 0.9999	
RA	48.0 ± 3.3	17.5 ± 3.0	69.6 ± 3.2	93.7 ± 1.5	65.8 ± 3.4	97.4 ± 0.8
	*p* = 0.0169 *		*p* = 0.2550		*p* = 0.0096 *	
Starvation	51.1 ± 3.2	48.1 ± 4.6	67.4 ± 5.0	71.0 ± 6.2	42.3 ± 3.6	48.1 ± 5.2
	*p* > 0.9999		*p* > 0.9999		*p* > 0.9999	

* Significant changes.

## Data Availability

The raw data supporting the conclusions of this article will be made available by the authors on request.

## References

[1] Biedler J.L., Helson L., Spengler B.A. (1973). Morphology and growth, tumorigenicity, and cytogenetics of human neuroblastoma cells in continuous culture. Cancer Res..

[2] Kovalevich J., Langford D. (2013). Considerations for the use of SH-SY5Y neuroblastoma cells in neurobiology. Methods Mol. Biol..

[3] Biedler J.L., Roffler-Tarlov S., Schachner M., Freedman L.S. (1978). Multiple neurotransmitter synthesis by human neuroblastoma cell lines and clones. Cancer Res..

[4] Oyarce A.M., Fleming P.J. (1991). Multiple forms of human dopamine beta-hydroxylase in SH-SY5Y neuroblastoma cells. Arch. Biochem. Biophys..

[5] Xie H.R., Hu L.S., Li G.Y. (2010). SH-SY5Y human neuroblastoma cell line: In vitro cell model of dopaminergic neurons in Parkinson’s disease. Chin. Med. J..

[6] Bell M., Zempel H. (2021). SH-SY5Y-derived neurons: A human neuronal model system for investigating TAU sorting and neuronal subtype-specific TAU vulnerability. Rev. Neurosci..

[7] Lopez-Suarez L., Awabdh S.A., Coumoul X., Chauvet C. (2022). The SH-SY5Y human neuroblastoma cell line, a relevant in vitro cell model for investigating neurotoxicology in human: Focus on organic pollutants. Neurotoxicology.

[8] Costa I., Barbosa D.J., Silva V., Benfeito S., Borges F., Remião F., Silva R. (2023). Research Models to Study Ferroptosis’s Impact in Neurodegenerative Diseases. Pharmaceutics.

[9] Ioghen O.C., Ceafalan L.C., Popescu B.O. (2023). SH-SY5Y Cell Line In Vitro Models for Parkinson Disease Research-Old Practice for New Trends. J. Integr. Neurosci..

[10] Pandey M., Karmakar V., Majie A., Dwivedi M., Md S., Gorain B. (2024). The SH-SY5Y cell line: A valuable tool for Parkinson’s disease drug discovery. Expert Opin. Drug Discov..

[11] Teppola H., Sarkanen J.R., Jalonen T.O., Linne M.L. (2016). Morphological Differentiation Towards Neuronal Phenotype of SH-SY5Y Neuroblastoma Cells by Estradiol, Retinoic Acid and Cholesterol. Neurochem. Res..

[12] Dos Santos M.G., Gomes J.R., Costa M.D.M. (2023). Methods used to achieve different levels of the neuronal differentiation process in SH-SY5Y and Neuro2a cell lines: An integrative review. Cell Biol. Int..

[13] Rastinejad F. (2001). Retinoid X receptor and its partners in the nuclear receptor family. Curr. Opin. Struct. Biol..

[14] Mangelsdorf D.J., Evans R.M. (1995). The RXR heterodimers and orphan receptors. Cell.

[15] Mangelsdorf D.J., Thummel C., Beato M., Herrlich P., Schütz G., Umesono K., Blumberg B., Kastner P., Mark M., Chambon P. (1995). The nuclear receptor superfamily: The second decade. Cell.

[16] Zhang T., Gygi S.P., Paulo J.A. (2021). Temporal Proteomic Profiling of SH-SY5Y Differentiation with Retinoic Acid Using FAIMS and Real-Time Searching. J. Proteome Res..

[17] Leung T.C.N., Lu S.N., Chu C.N., Lee J., Liu X., Ngai S.M. (2024). Temporal Quantitative Proteomic and Phosphoproteomic Profiling of SH-SY5Y and IMR-32 Neuroblastoma Cells during All-*Trans*-Retinoic Acid-Induced Neuronal Differentiation. Int. J. Mol. Sci..

[18] López-Carballo G., Moreno L., Masia S., Perez P., Barettino D. (2002). Activation of the phosphatidylinositol 3-kinase/Akt signaling pathway by retinoic acid is required for neural differentiation of SH-SY5Y human neuroblastoma cells. J. Biol. Chem..

[19] Janesick A., Wu S.C., Blumberg B. (2015). Retinoic acid signaling and neuronal differentiation. Cell. Mol. Life Sci..

[20] Watanabe K., Yamaji R., Ohtsuki T. (2018). MicroRNA-664a-5p promotes neuronal differentiation of SH-SY5Y cells. Genes Cells.

[21] Dutta S., Pal D., Rao M.R.S. (2024). Retinoic Acid-Mediated Differentiation of Mouse Embryonic Stem Cells to Neuronal Cells. Methods Mol. Biol..

[22] Encinas M., Iglesias M., Liu Y., Wang H., Muhaisen A., Ceña V., Gallego C., Comella J.X. (2000). Sequential treatment of SH-SY5Y cells with retinoic acid and brain derived neurotrophic factor gives rise to fully differentiated, neurotrophic factor dependent, human neuron-like cells. J. Neurochem..

[23] Edsjö A., Holmquist L., Påhlman S. (2007). Neuroblastoma as an experimental model for neuronal differentiation and hypoxia-induced tumor cell dedifferentiation. Semin. Cancer Biol..

[24] Zeng M., Zhou J.N. (2008). Role of autophagy and mTOR signaling in neuronal differentiation of mouse neuroblastoma cells. Cell. Signal..

[25] Nematisouldaragh D., Kirshenbaum E., Uzonna M., Kirshenbaum L., Rabinovich-Nikitin I. (2024). The Role of Retinoic-Acid-Related Orphan Receptor (RRs) in Cellular Homeostasis. Int. J. Mol. Sci..

[26] Klionsky D.J., Emr S.D. (2000). Autophagy as a regulated pathway of cellular degradation. Science.

[27] Kim E., Goraksha-Hicks P., Li L., Neufeld T.P., Guan K.L. (2008). Regulation of TORC1 by Rag GTPases in nutrient response. Nat. Cell Biol..

[28] Sancak Y., Peterson T.R., Shaul Y.D., Lindquist R.A., Thoreen C.C., Bar-Peled L., Sabatini D.M. (2008). The Rag GTPases bind raptor and mediate amino acid signaling to mTORC1. Science.

[29] Sancak Y., Bar-Peled L., Zoncu R., Markhard A.L., Nada S., Sabatini D.M. (2010). Ragulator-Rag complex targets mTORC1 to the lysosomal surface and is necessary for its activation by amino acids. Cell.

[30] Nowosad A., Jeannot P., Callot C., Creff J., Perchey R.T., Joffre C., Codogno P., Manenti S., Besson A. (2020). p27 controls Ragulator and mTOR activity in amino acid-deprived cells to regulate the autophagy-lysosomal pathway and coordinate cell cycle and cell growth. Nat. Cell Biol..

[31] Li X., Wang S., Yu X., Li S. (2023). Transcriptional regulation of autophagy by chromatin remodeling complex and histone variant. Autophagy.

[32] Ashrafi G., Schwarz T.L. (2013). The pathways of mitophagy for quality control and clearance of mitochondria. Cell Death Differ..

[33] Yang M., Luo S., Wang X., Li C., Yang J., Zhu X., Xiao L., Sun L. (2021). ER-Phagy: A New Regulator of ER Homeostasis. Front. Cell Dev. Biol..

[34] Lu L.Q., Tang M.Z., Qi Z.H., Huang S.F., He Y.Q., Li D.K., Li L.F., Chen L.X. (2020). Regulation of the Golgi apparatus via GOLPH3-mediated new selective autophagy. Life Sci..

[35] Kuma A., Mizushima N. (2010). Physiological role of autophagy as an intracellular recycling system: With an emphasis on nutrient metabolism. Semin. Cell Dev. Biol..

[36] Singh R., Cuervo A.M. (2011). Autophagy in the cellular energetic balance. Cell Metab..

[37] Kaur J., Debnath J. (2015). Autophagy at the crossroads of catabolism and anabolism. Nat. Rev. Mol. Cell Biol..

[38] Choi A.M., Ryter S.W., Levine B. (2013). Autophagy in human health and disease. N. Engl. J. Med..

[39] Mizushima N. (2018). A brief history of autophagy from cell biology to physiology and disease. Nat. Cell Biol..

[40] Deretic V., Kroemer G. (2021). Autophagy in metabolism and quality control: Opposing, complementary or interlinked functions?. Autophagy.

[41] Domínguez-Martín H., Gavilán E., Parrado C., Burguillos M.A., Daza P., Ruano D. (2024). Distinct UPR and Autophagic Functions Define Cell-Specific Responses to Proteotoxic Stress in Microglial and Neuronal Cell Lines. Cells.

[42] Juhasz G., Csikos G., Sinka R., Erdelyi M., Sass M. (2003). The Drosophila homolog of Aut1 is essential for autophagy and development. FEBS Lett..

[43] Wu X., Fleming A., Ricketts T., Pavel M., Virgin H., Menzies F.M., Rubinsztein D.C. (2016). Autophagy regulates Notch degradation and modulates stem cell development and neurogenesis. Nat. Commun..

[44] Ferrucci M., Biagioni F., Lenzi P., Gambardella S., Ferese R., Calierno M.T., Falleni A., Grimaldi A., Frati A., Esposito V. (2017). Rapamycin promotes differentiation increasing βIII-tubulin, NeuN, and NeuroD while suppressing nestin expression in glioblastoma cells. Oncotarget.

[45] Manzoli R., Badenetti L., Rubin M., Moro E. (2021). Lysosomal Function and Axon Guidance: Is There a Meaningful Liaison?. Biomolecules.

[46] Lewerissa E.I., Nadif Kasri N., Linda K. (2024). Epigenetic regulation of *autophagy-related* genes: Implications for neurodevelopmental disorders. Autophagy.

[47] Liénard C., Pintart A., Bomont P. (2024). Neuronal Autophagy: Regulations and Implications in Health and Disease. Cells.

[48] Restrepo L.J., Baehrecke E.H. (2024). Regulation and Functions of Autophagy During Animal Development. J. Mol. Biol..

[49] Xu L., Saeed S., Ma X., Cen X., Sun Y., Tian Y., Zhang D., Tang A., Zhou H., Lai J. (2024). Hippocampal mitophagy contributes to spatial memory via maintaining neurogenesis during the development of mice. CNS Neurosci. Ther..

[50] Shen W., Ganetzky B. (2009). Autophagy promotes synapse development in Drosophila. J. Cell Biol..

[51] Vessoni A.T., Muotri A.R., Okamoto O.K. (2012). Autophagy in stem cell maintenance and differentiation. Stem Cells Dev..

[52] Tang G., Gudsnuk K., Kuo S.H., Cotrina M.L., Rosoklija G., Sosunov A., Sonders M.S., Kanter E., Castagna C., Yamamoto A. (2014). Loss of mTOR-dependent macroautophagy causes autistic-like synaptic pruning deficits. Neuron.

[53] Yamaguchi J., Suzuki C., Nanao T., Kakuta S., Ozawa K., Tanida I., Saitoh T., Sunabori T., Komatsu M., Tanaka K. (2018). Atg9a deficiency causes axon-specific lesions including neuronal circuit dysgenesis. Autophagy.

[54] Stavoe A.K.H., Holzbaur E.L.F. (2019). Autophagy in Neurons. Annu. Rev. Cell Dev. Biol..

[55] Hassan B.A., Hiesinger P.R. (2023). Autophagy in synapse formation and brain wiring. Autophagy.

[56] Komatsu M., Waguri S., Chiba T., Murata S., Iwata J., Tanida I., Ueno T., Koike M., Uchiyama Y., Kominami E. (2006). Loss of autophagy in the central nervous system causes neurodegeneration in mice. Nature.

[57] Komatsu M., Wang Q.J., Holstein G.R., Friedrich V.L., Iwata J., Kominami E., Chait B.T., Tanaka K., Yue Z. (2007). Essential role for autophagy protein Atg7 in the maintenance of axonal homeostasis and the prevention of axonal degeneration. Proc. Natl. Acad. Sci. USA.

[58] Akizu N., Cantagrel V., Zaki M.S., Al-Gazali L., Wang X., Rosti R.O., Dikoglu E., Gelot A.B., Rosti B., Vaux K.K. (2015). Biallelic mutations in SNX14 cause a syndromic form of cerebellar atrophy and lysosome-autophagosome dysfunction. Nat. Genet..

[59] Zhao Y.G., Sun L., Miao G., Ji C., Zhao H., Sun H., Miao L., Yoshii S.R., Mizushima N., Wang X. (2015). The autophagy gene Wdr45/Wipi4 regulates learning and memory function and axonal homeostasis. Autophagy.

[60] Kim M., Sandford E., Gatica D., Qiu Y., Liu X., Zheng Y., Schulman B.A., Xu J., Semple I., Ro S.H. (2016). Mutation in ATG5 reduces autophagy and leads to ataxia with developmental delay. Elife.

[61] Hori I., Otomo T., Nakashima M., Miya F., Negishi Y., Shiraishi H., Nonoda Y., Magara S., Tohyama J., Okamoto N. (2017). Defects in autophagosome-lysosome fusion underlie Vici syndrome, a neurodevelopmental disorder with multisystem involvement. Sci. Rep..

[62] Collier J.J., Guissart C., Oláhová M., Sasorith S., Piron-Prunier F., Suomi F., Zhang D., Martinez-Lopez N., Leboucq N., Bahr A. (2021). Developmental consequences of defective ATG7-mediated autophagy in humans. N. Engl. J. Med..

[63] Lei Y., Klionsky D.J. (2021). The Emerging Roles of Autophagy in Human Diseases. Biomedicines.

[64] Deneubourg C., Ramm M., Smith L.J., Baron O., Singh K., Byrne S.C., Duchen M.R., Gautel M., Eskelinen E.L., Fanto M. (2022). The spectrum of neurodevelopmental, neuromuscular and neurodegenerative disorders due to defective autophagy. Autophagy.

[65] Klionsky D.J., Cuervo A.M., Seglen P.O. (2007). Methods for monitoring autophagy from yeast to human. Autophagy.

[66] Xie Z., Klionsky D.J. (2007). Autophagosome formation: Core machinery and adaptations. Nat. Cell Biol..

[67] Feng Y., He D., Yao Z., Klionsky D.J. (2014). The machinery of macroautophagy. Cell Res..

[68] Yin Z., Pascual C., Klionsky D.J. (2016). Autophagy: Machinery and regulation. Microb. Cell.

[69] Yang Z.F., Klionsky D.J. (2010). Eaten alive: A history of Macroautophagy. Nat. Cell Biol..

[70] Wang Y., Gao J., Fan B., Hu Y., Yang Y., Wu Y., Li F., Ju H. (2023). Different levels of autophagy induced by transient serum starvation regulate metabolism and differentiation of porcine skeletal muscle satellite cells. Sci. Rep..

[71] Tanida I., Ueno T., Kominami E. (2004). LC3 conjugation system in mammalian autophagy. Int. J. Biochem. Cell Biol..

[72] Tanida I., Ueno T., Kominami E. (2008). LC3 and Autophagy. Methods Mol. Biol..

[73] Cowley S., Paterson H., Kemp P., Marshall C.J. (1994). Activation of MAP kinase kinase is necessary and sufficient for PC12 differentiation and for transformation of NIH 3T3 cells. Cell.

[74] Ganley I.G., Lam D.H., Wang J., Ding X., Chen S., Jiang X. (2009). ULK1.ATG13.FIP200 complex mediates mTOR signaling and is essential for autophagy. J. Biol. Chem..

[75] Laplante M., Sabatini D.M. (2009). mTOR signaling at a glance. J. Cell Sci..

[76] Kim J., Kundu M., Viollet B., Guan K.L. (2011). AMPK and mTOR regulate autophagy through direct phosphorylation of Ulk1. Nat. Cell Biol..

[77] Saxton R.A., Sabatini D.M. (2017). mTOR signaling in growth, metabolism, and disease. Cell.

[78] Skop V., Cahova M., Dankova H., Papackova Z., Palenickova E., Svoboda P., Zidkova J., Kazdova L. (2014). Autophagy inhibition in early but not in later stages prevents 3T3-L1 differentiation: Effect on mitochondrial remodeling. Differentiation.

[79] Carpentieri A., Cozzoli E., Scimeca M., Bonanno E., Sardanelli A.M., Gambacurta A. (2016). Differentiation of human neuroblastoma cells toward the osteogenic lineage by mTOR inhibitor. Cell Death. Dis..

[80] Sidell N. (1982). Retinoic acid-induced growth inhibition and morphologic differentiation of human neuroblastoma cells in vitro. J. Natl. Cancer Inst..

[81] Påhlman S., Ruusala A., Abrahamsson L., Mattsson M.E., Esscher T. (1984). Retinoic acid-induced differentiation of cultured human neuroblastoma cells: A comparison with phorbolester-induced differentiation. Cell Differ..

[82] Påhlman S., Hoehner J.C., Nånberg E., Hedborg F., Fagerström S., Gestblom C., Johansson I., Larsson U., Lavenius E., Ortoft E. (1995). Differentiation and survival influences of growth factors in human neuroblastoma. Eur. J. Cancer.

[83] Cheung Y.T., Lau W.K., Yu M.S., Lai C.S., Yeung S.C., So K.F., Chang R.C. (2009). Effects of all-trans-retinoic acid on human SH-SY5Y neuroblastoma as in vitro model in neurotoxicity research. Neurotoxicology.

[84] Harasym E., McAndrew N., Gomez G. (2017). Sub-micromolar concentrations of retinoic acid induce morphological and functional neuronal phenotypes in SK-N-SH neuroblastoma cells. Vitr. Cell. Dev. Biol.-Anim..

[85] Encinas M., Iglesias M., Llecha N., Comella J.X. (1999). Extracellular-regulated kinases and phosphatidylinositol 3-kinase are involved in brain-derived neurotrophic factor-mediated survival and neuritogenesis of the neuroblastoma cell line SH-SY5Y. J. Neurochem..

[86] Agholme L., Lindström T., Kågedal K., Marcusson J., Hallbeck M. (2010). An in vitro model for neuroscience: Differentiation oh SHSY5Y cells into cells with morphological and biochemical characteristics of mature neurons. J. Alzheimer’s Dis..

[87] Lopes F.M., Schröder R., da Frota M.L.C., Zanotto-Filho A., Müller C.B., Pires A.S., Meurer R.T., Colpo G.D., Gelain D.P., Kapczinski F. (2010). Comparison between proliferative and neuron-like SH-SY5Y cells as an in vitro model for Parkinson disease studies. Brain Res..

[88] Simões R.F., Ferrão R., Silva M.R., Pinho S.L.C., Ferreira L., Oliveria P.J., Cunha-Oliveira T. (2021). Refinement of a differentiation protocol using neuroblastoma SH-SY5Y cells for use in neurotoxicology research. Food Chem. Toxicol..

[89] Melino G., Thiele C.J., Knight R.A., Piacentini M. (1997). Retinoids and the control of growth/death decisions in human neuroblastoma cell lines. J. Neuro-Oncol..

[90] Pogenberg V., Guichou J.F., Vivat-Hannah V., Kammerer S., Perez E., Germain P., de Lera A.R., Gronemeyer H., Royer C.A., Bourguet W. (2005). Characterization of the interaction between retinoic acid receptor/retinoid X receptor (RAR/RXR) heterodimers and transcriptional coactivators through structural and fluorescence anisotropy studies. J. Biol. Chem..

[91] Joshi S., Guleria R., Pan J., DiPette D., Singh U.S. (2006). Retinoic acidreceptors and tissue-transglutaminase mediate short-termeffect of retinoic acid on migration and invasion ofneuroblastoma SH-SY5Y cells. Oncogene.

[92] Borsani E., Buffoli B., Bonazza V., Brunelli G., Monini L., Inchingolo F., Ballini A., Rezzani R., Rodella L.F. (2020). In vitro effects of concentrated growth factors (CGF) on human SH-SY5Y neuronal cells. Eur. Rev. Med. Pharmacol. Sci..

[93] Itano Y., Ito A., Uehara T., Nomura Y. (1996). Regulation of Bcl-2 protein expression in human neuroblastoma SH-SY5Y cells: Positive and negative effects of protein kinases C and A, respectively. J. Neurochem..

[94] Tieu K., Zuo D.M., Yu P.H. (1999). Differential effects of staurosporine and retinoic acid on the vulnerability of the SH-SY5Y neuroblastoma cells: Involvement of bcl-2 and p53 proteins. J. Neurosci. Res..

[95] Dodurga Y., Gundogdu G., Koc T., Yonguc G.N., Kucukatay V., Satiroglu-Tufan N.L. (2013). Expression of URG4/URGCP, Cyclin D1, Bcl-2, and Bax genes in retinoic acid treated SH-SY5Y human neuroblastoma cells. Contemp. Oncol..

[96] Xu H.D., Wu D., Gu J.H., Ge J.B., Wu J.C., Han R., Liang Z.Q., Qin Z.H. (2013). The pro-survival role of autophagy depends on Bcl-2 under nutrition stress conditions. PLoS ONE.

[97] Shipley M.M., Mangold C.A., Szpara M.L. (2016). Differentiation of the SH-SY5Y Human Neuroblastoma Cell Line. J. Vis. Exp..

[98] Seglen P.O., Gordon P.B. (1982). 3-Methyladenine: Specific inhibitor of autophagic/lysosomal protein degradation in isolated rat hepatocytes. Proc. Natl. Acad. Sci. USA.

[99] Raught B., Gingras A.C., Sonenberg N. (2001). The target of rapamycin (TOR) proteins. Proc. Natl. Acad. Sci. USA.

[100] Castino R., Lazzeri G., Lenzi P., Bellio N., Follo C., Ferrucci M., Fornai F., Isidoro C. (2008). Suppression of autophagy precipitates neuronal cell death following low doses of methamphetamine. J. Neurochem..

[101] Fornai F., Longone P., Ferrucci M., Lenzi P., Isidoro C., Ruggieri S., Paparelli A. (2008). Autophagy and amyotrophic lateral sclerosis: The multiple roles of lithium. Autophagy.

[102] Lazzeri G., Biagioni F., Fulceri F., Busceti C.L., Scavuzzo M.C., Ippolito C., Salvetti A., Lenzi P., Fornai F. (2018). mTOR Modulates Methamphetamine-Induced Toxicity through Cell Clearing Systems. Oxid. Med. Cell Longev..

[103] Ferese R., Lenzi P., Fulceri F., Biagioni F., Fabrizi C., Gambardella S., Familiari P., Frati A., Limanaqi F., Fornai F. (2020). Quantitative Ultrastructural Morphometry and Gene Expression of mTOR-Related Mitochondriogenesis within Glioblastoma Cells. Int. J. Mol. Sci..

[104] Ferrucci M., Busceti C.L., Lazzeri G., Biagioni F., Puglisi-Allegra S., Frati A., Lenzi P., Fornai F. (2022). Bacopa Protects against Neurotoxicity Induced by MPP+ and Methamphetamine. Molecules.

[105] Scholzen T., Gerdes J. (2000). The Ki-67 protein: From the known and the unknown. Cell Physiol..

[106] Kee N., Sivalingam S., Boonstra R., Wojtowicz J.M. (2002). The utility of Ki-67 and BrdU as proliferative markers of adult neurogenesis. J. Neurosci. Methods.

[107] Lendahl U., Zimmerman L.B., McKay R.D.G. (1990). CNS stem cells express a new class of intermediate filament protein. Cell.

[108] Menezes J.R., Luskin M.B. (1994). Expression of neuron-specific tubulin defines a novel population in the proliferative layers of the developing telencephalon. J. Neurosci..

[109] Bédard A., Parent A. (2004). Evidence of newly generated neurons in the human olfactory bulb. Brain Res. Dev. Brain Res..

[110] Mullen R.J., Buck C.R., Smith A.M. (1992). NeuN, a neuronal specific nuclear protein in vertebrates. Development.

[111] Liang X.H., Jackson S., Seaman M., Brown K., Kempkes B., Hibshoosh H., Levine B. (1999). Induction of autophagy and inhibition of tumorigenesis by beclin 1. Nature.

[112] Anwar T., Liu X., Suntio T., Marjamäki A., Biazik J., Chan E.Y.W., Varjosalo M., Eskelinen E.L. (2019). ER-Targeted Beclin 1 Supports Autophagosome Biogenesis in the Absence of ULK1 and ULK2 Kinases. Cells.

[113] Hill S.M., Wrobel L., Ashkenazi A., Fernandez-Estevez M., Tan K., Bürli R.W., Rubinsztein D.C. (2021). VCP/p97 regulates Beclin-1-dependent autophagy initiation. Nat. Chem. Biol..

[114] Klionsky D.J., Abdel-Aziz A.K., Abdelfatah S., Abdellatif M., Abdoli A., Abel S., Abeliovich H., Abildgaard M.H., Abudu Y.P., Acevedo-Arozena A. (2021). Guidelines for the use and interpretation of assays for monitoring autophagy (4th edition). Autophagy.

[115] Marwaha R., Sharma M. (2017). DQ-Red BSA Trafficking Assay in Cultured Cells to Assess Cargo Delivery to Lysosomes. Bio Protoc..

[116] Bendayan M., Zollinger M. (1983). Ultrastructural localization of antigenic sites on osmium-fixed tissues applying the protein A-gold technique. J. Histochem. Cytochem..

[117] Lenzi P., Marongiu R., Falleni A., Gelmetti V., Busceti C.L., Michiorri S., Valente E.M., Fornai F.A. (2012). A subcellular analysis of genetic modulation of PINK1 on mitochondrial alterations, autophagy and cell death. Arch. Ital. Biol..

[118] Lucocq J.M., Habermann A., Watt S., Backer J.M., Mayhew T.M., Griffiths G. (2004). A rapid method for assessing the distribution of gold labeling on thin sections. J. Histochem. Cytochem..

[119] Lenzi P., Lazzeri G., Biagioni F., Busceti C.L., Gambardella S., Salvetti A., Fornai F. (2016). The autophagoproteasome a novel cell clearing organelle in baseline and stimulated conditions. Front. Neuroanat..

